# A chaperonin BnaC01.CCT8 contributes to silique length and seed weight by affecting auxin and jasmonic acid signalling in *Brassica napus*

**DOI:** 10.1111/pbi.70184

**Published:** 2025-06-18

**Authors:** Zhaoyang Qu, Ze Tian, Liqing Wei, Chao Wang, Lieqiong Kuang, Jiaqi Yan, Furong Wang, Nian Wang, Jinxing Tu, Xinfa Wang, Hanzhong Wang, Xiaoling Dun

**Affiliations:** ^1^ Key Laboratory of Biology and Genetic Improvement of Oil Crops, Oil Crops Research Institute of the Chinese Academy of Agricultural Sciences Ministry of Agriculture and Rural Affairs Wuhan China; ^2^ National Key Laboratory of Crop Genetic Improvement Huazhong Agricultural University Wuhan China; ^3^ College of Agriculture Northwest A&F University Yangling China; ^4^ Hubei Hongshan Laboratory Wuhan China

**Keywords:** silique length, seed weight, auxin, jasmonic acid, *BnaC01.CCT8*, CCT complex

## Abstract

Seed weight (SW), which is directly influenced by silique length (SL), is a critical agronomic trait significantly affecting both the quality and yield of rapeseed. In this study, a *shorter silique length* (*ssl*) mutant was generated through ethyl methane sulfonate mutagenesis, exhibiting reduced SL and SW compared to the wild type. Utilizing a map‐based cloning approach, *BnaC01.CCT8*, a member of the chaperonin containing T‐complex polypeptide‐1 (CCT) family, was identified as the gene responsible for restoring the *ssl* phenotype. A missense mutation from alanine to valine (A507V) in *BnaC01.CCT8* was identified as crucial for its functional activity, as evidenced by the genetic complementation of *BnaC01.CCT8* and *BnaC01.CCT8* ^A507V^ in the Arabidopsis *cct8‐2* background. Moreover, overexpression of *BnaC01.CCT8* in *Brassica napus* significantly enhanced SL, SW and seed yield per plant. Conversely, CRISPR/Cas9‐mediated *bnac01.cct8* knockout lines exhibited reduced SL and SW. Transcriptome analysis and hormone content detection indicated that *BnaC01.CCT8* positively regulated SL and SW primarily by modulating auxin and jasmonic acid signalling, thereby affecting the length of epidermal cells in the silique wall. Furthermore, BnaC01.CCT8 interacted with BnaA09.ARF18 (AUXIN‐RESPONSE FACTOR 18), contributing to the regulation of SL and SW, while the A507V mutation disrupted this interaction. Haplotype analysis demonstrated that several SNP differences in *BnaC01.CCT8* were significantly associated with variations in SL and SW among germplasm resources, revealing superior alleles of *BnaC01.CCT8*. The identification and functional analysis of *BnaC01.CCT8* provide new insights into the mechanisms regulating SL and SW and present a valuable target for the genetic enhancement of rapeseed yield.

## Introduction

The continuous improvement of crop yield remains an enduring objective in agricultural production. Seed weight (SW) is recognized as one of the three primary factors influencing grain yield (Qi *et al*., 2013; Raboanatahiry *et al*., 2018; Wang *et al*., 2024). Rapeseed (*Brassica napus* L.) ranks as the third largest oil‐producing crop globally, providing essential edible oil for human consumption. Siliques function as both vital storage and photosynthetic organs in rapeseed, supplying crucial nutrients for seed development while also protecting seeds from biological and abiotic stresses (Li *et al*., 2018; Zhu *et al*., 2018). Longer siliques create an expanded space and enhance nutritional support for developing seeds, potentially resulting in either larger seeds or an increased seed number per silique (SPS) (Bennett *et al*., 2011; Chay and Thurling, 2010).

Both SW and silique length (SL) are widely recognized as complex traits governed by multiple genes (Chen *et al*., 2007; Qi *et al*., 2013; Yang *et al*., 2012; Zhang *et al*., 2011). Numerous pleiotropic quantitative trait loci (QTL) that concurrently regulate both SW and SL have been identified across chromosomes A01, A02, A05, A09, C01, C03, C05, C06, C07 and C08 in *B. napus* (Hu *et al*., 2022; Raboanatahiry *et al*., 2018). However, the cloning of key regulators for SL and SW has been limited, with only a few regulatory genes discovered. For instance, *BnaA09.ARF18* (*AUXIN‐RESPONSE FACTOR 18*), the first identified QTL for SL and SW in rapeseed, mediates the cell growth of the silique wall through an auxin‐response pathway, thereby affecting SW via maternal effects (Liu *et al*., 2015). *BnaA09.CYP78A9* (*CYTOCHROME P450 78A9*) encodes a P450 monooxygenase that positively regulates auxin content, promoting both silique elongation and seed expansion (Shen *et al*., 2019; Shi *et al*., 2019; Ye *et al*., 2023). Genome‐wide association studies (GWAS) have identified *BnaA02.SE* (*SILIQUE ELONGATION*) as a key modulator of cell proliferation and expansion in siliques through the regulation of jasmonic acid (JA) and indole‐3‐acetic acid (IAA) levels, ultimately enhancing SW (Zhang *et al*., 2024). Additionally, studies on homologues of Arabidopsis genes have identified *BnaA05.DAD1* (*DEFECTIVE IN ANTHER DEHISCENCE 1*) and *BnaEOD3s* (*ENHANCER3 OF DA1/CYP78A6*) as positive regulators of SW and SL in *B. napus* (Khan *et al*., 2020; Liu *et al*., 2021).

Furthermore, several genes have been identified that independently regulate SL and SW. Through associative transcriptomics, *BnaC03.UPL3* (*UBIQUITIN‐PROTEIN LIGASE 3*) was found to exhibit a negative correlation with SW (Miller *et al*., 2019). *BnaARF2s* (*AUXIN‐RESPONSE FACTOR 2*) have been identified as negative regulators of SW, while *BnaMPK3s* have demonstrated conserved functions in promoting organ size through their interaction with *BnaARF2s* (Tian *et al*., 2023). Additionally, several genes, including *BnaGRF2a* (*GROWTH‐REGULATING FACTOR 2‐LIKE GENE*), *BnaCLV3s* (*CLAVATA3*) and *BnaUBP15s* (*UBIQUITIN‐SPECIFIC PROTEASE 15*), have been reported to positively influence SW (Gu *et al*., 2023; Khan *et al*., 2020; Liu *et al*., 2012, 2021; Yang *et al*., 2018). *BnaC07.ROT3* (*ROTUNDIFOLIA3*) is critical for brassinosteroid (BR) biosynthesis and enhances SL by promoting cell elongation in the silique wall (Zhou *et al*., 2022). Previous research has demonstrated that plant hormones, particularly auxin, are essential for silique and seed development. Elevated auxin concentrations may stimulate cell growth, leading to increased SL and ultimately contributing to a rise in SW (Shen *et al*., 2019; Shi *et al*., 2019; Ye *et al*., 2023; Zhang *et al*., 2024). Therefore, identifying auxin‐related genes that regulate SL and SW is crucial for enhancing rapeseed yield.

The chaperonin containing T‐complex polypeptide‐1 (CCT) is an evolutionarily conserved cytosolic group II chaperonin found in all eukaryotes (Dekker *et al*., 2008; Yébenes *et al*., 2011). The CCT complex comprises eight homologous subunits arranged in a specific order, forming a cage‐like structure (CCT 1–4–2‐5‐7‐8‐6‐3) (Joachimiak *et al*., 2014; Leitner *et al*., 2012). Sequence variations among the eight CCT subunits are primarily localized within their apical domains, which confer substrate binding specificity to each subunit (Spiess *et al*., 2006). The CCT complex facilitates the folding of approximately 10% of cellular proteins into their active states by encapsulating unfolded substrate proteins within its central cavities (Cong *et al*., 2012; Horwich *et al*., 2007; Noormohammadi *et al*., 2016). Furthermore, the CCT complex is essential for maintaining telomeres and organizing actin and tubulin structures (Freund *et al*., 2014). Notably, the loss of a single CCT subunit disrupts the assembly and function of the CCT complex; however, the expression of the key activator subunit CCT8 is sufficient to promote the assembly of the entire complex and enhance the quality of the folded proteins (Grantham, 2020; Noormohammadi *et al*., 2016). In *Arabidopsis*, nine genes encode eight distinct chaperonin subunits (CCT1‐8), which are crucial for plant growth and development (Ahn *et al*., 2019). The overexpression of *CCT8* alleviates protein aggregation in differentiated cells and confers resistance to proteotoxic stress (Llamas *et al*., 2021). CCT8 functions synergistically with RRP44A (Ribosomal RNA‐Processing Protein 44) to mediate the cell‐to‐cell trafficking of *KN1* (*KNOTTED1*) mRNA and protein via plasmodesmata, contributing to the establishment and/or maintenance of stem cells (Kitagawa *et al*., 2022; Xu *et al*., 2011). Additionally, CCT8 facilitates the intercellular movement of viruses and the long‐distance transport of ribonucleoprotein complexes (Fichtenbauer *et al*., 2012; Wang *et al*., 2023). However, the essential roles of CCT in cell survival complicate the acquisition of loss‐of‐function mutants, thereby hindering research in plants. The molecular mechanisms through which CCT family genes regulate the plant hormone signalling pathway remain unclear.

In this study, a *short silique length* (*ssl*) mutant characterized by reduced SL and SW was isolated through ethyl methane sulfonate (EMS) mutagenesis. The gene responsible for the *ssl* phenotype was successfully identified from the CCT family and designated as *BnaC01.CCT8*. Through overexpression, CRISPR/Cas9, and genetic complementation, this study established the positive regulatory role of *BnaC01.CCT8* in both SL and SW. Transcriptome analysis, quantitative reverse transcription PCR (qRT‐PCR), hormone content detection, and protein interaction analysis indicated that *BnaC01.CCT8* primarily regulated SL and SW through auxin and JA signalling pathways. Haplotype analysis revealed superior alleles of *BnaC01.CCT8* that enhanced both SL and SW. This research provides new insights into the molecular mechanisms underlying silique and seed development, while also offering valuable genetic resources for high‐yield breeding in rapeseed.

## Results

### Genetic mapping of the causal gene for the *ssl* mutant

**Figure 1:**
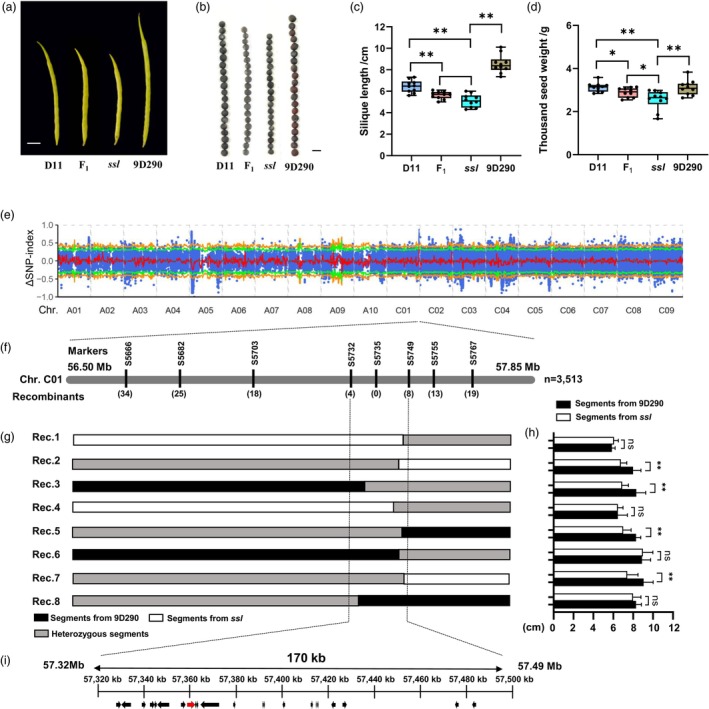
Genetic mapping of the *ssl* gene. (a, b) The SL (a) and SW (b) phenotypes of D11 (WT), F_1_ (D11 × *ssl*), *ssl* and 9D290. Scale bar = 1 cm in (a) and 2 mm in (b). (c, d) Statistical comparison of SL and TSW in (a) and (b), respectively. (e) Initial mapping of the *ssl* locus on chromosome C01 by BSA. (f) Fine mapping of the *ssl* locus. Numbers in parentheses indicate the number of recombinants detected by the corresponding marker. (g) Genotypes of the representative recombinants by progeny test. Black, white and grey bars indicate the homozygous target segments from 9D290, *ssl* and heterozygous segments, respectively. (h) Statistical comparison of SL of progeny from the representative recombinants in (g). (i) Candidate genes in the mapped 170 kb of the *ssl* locus annotated by the ‘ZS11v0’ genome (Song *et al*., 2020). Annotated genes are represented as arrows, indicating the direction of transcription. Red arrow denotes the candidate gene, *BnaC01G0509300ZS*. ns, *p* > 0.05; *, *p* < 0.05; **, *p* < 0.01; ***, *p* < 0.001.

The *ssl* mutant was generated through EMS mutagenesis of D11, a semi‐winter inbred line of *B. napus*. Compared to the wild type (WT, D11), both SL and thousand seed weight (TSW) in the *ssl* mutant exhibited significant reductions at maturity (Figure 1a–d). The F_1_ progeny resulting from the cross between D11 and *ssl* displayed intermediate values of SL and TSW relative to their parental lines (Figure 1a–d). Therefore, we proposed that the phenotype of *ssl* was governed by a semidominant gene. To fine‐map the *ssl* locus, a segregating population was constructed by hybridizing a longer SL inbred line 9D290 (8.53 ± 1.77 cm) with *ssl* (5.20 ± 1.28 cm) (Figure 1a,c). A significant difference in TSW was also observed between 9D290 (3.11 ± 0.72 g) and *ssl* (2.54 ± 0.88 g) (Figure 1b,d). Given the ease of evaluation, SL was utilized for map‐based cloning of *ssl*. The SL of 316 individuals from line L120, derived from the F_2:5_ population, exhibited a normal distribution (Figure S1a). Furthermore, a bulked segregant analysis (BSA) was conducted using two pools: one longer SL and another for shorter SL, both derived from the 316 individuals. Consequently, a candidate interval for the *ssl* locus was identified on chromosome C01, spanning from 56.50 to 57.85 Mb, with a 99% confidence level (Figure 1e).

The near‐isogenic lines (NIL), NIL‐9D290 and NIL‐*ssl*, were developed from the progeny of line L120. These lines exhibited significant differences in SL and TSW, while no notable variations were detected in other agronomic traits, including seed number per pod, branch number, plant height, and dry weight (Figure S1b). Utilizing single nucleotide polymorphisms (SNPs) within the candidate interval, eight PARMS markers (Table S1) were developed for genotyping 3513 individuals in the F_2:6_ population derived from line L120 (Figure 1f). Through progeny testing of recombinant plants, the *ssl* locus was fine‐mapped to a physical interval of 170 kb, encompassing 22 annotated genes (Figure 1g–i).

### Expression pattern and subcellular localization of *BnaC01* *.* *CCT8*

**Figure 2:**
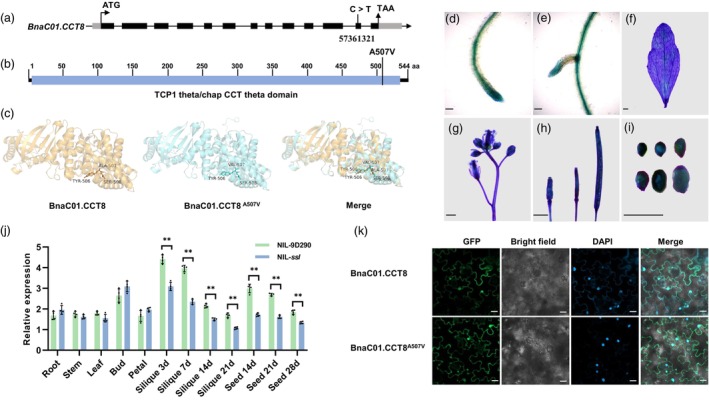
Variation, expression patterns and subcellular localization of *BnaC01.CCT8*. (a) Gene structure of *BnaC01.CCT8*. The mutation of *BnaC01.CCT8* in *ssl* is indicated by C > T in the 12th exon. Black bars, exons; lines, introns; grey bars, untranslated regions; ATG, start codon; TAA, stop codon. (b) Protein structure of BnaC01.CCT8. The amino acid (aa) substitution from alanine (Ala) to valine (Val) is highlighted with A507V. Blue bars indicate the TCP1 theta/chap CCT theta conserved domain. Numbers denote the aa position. (c) Three‐dimensional structure prediction and comparison of BnaC01.CCT8 and BnaC01.CCT8^A507V^. The locations and conformations of BnaC01.CCT8 and BnaC01.CCT8^A507V^ are merged for comparison. (d‐i) GUS staining of Arabidopsis tissues heterologously introduced with *pBnaC01.CCT8::GUS*, including root tip (d), lateral root (e), leaf (f), stems (g), inflorescence (g), siliques (h) and seeds (i). Scale bar = 100 μm in (d), (e) and (i). Scale bar = 2 mm in (f), (g) and (h). (j) qRT‐qPCR analysis of *BnaC01.CCT8* in various tissues of NIL‐9D290 and NIL‐*ssl*. 3d, 7d, 14d, 21d and 28d represent 3, 7, 14, 21 and 28 days after flowering, respectively. *BnaC02.ACTIN7* was used as a control. **, *p* < 0.01. (k) The subcellular localization of BnaC01.CCT8‐GFP and BnaC01.CCT8^A507V^‐GFP fusion proteins in tobacco. DAPI, a nuclear dye. Merge, a combination of GFP signal, bright field and DAPI staining. Scale bar = 20 μm.

Based on whole‐genome resequencing of D11 and *ssl*, a SNP (C to T) at position 57 361 321 in exon 12 of *BnaC01G0509300ZS* (*BnaC01.CCT8*) was identified, leading to an amino acid substitution from alanine to valine (A507V) within the predicted α‐helix structure of the TCP1 theta/chap CCT theta domain (Figure 2a–c). Previous research has demonstrated that an analogous amino acid substitution (A512V) in the mutant *atcct8‐1* occurred at a highly conserved residue, resulting in suppressed plant growth in *Arabidopsis* (Xu *et al*., 2011). Sequence alignment further revealed that both the *ssl* and *atcct8‐1* mutations affected the same amino acid site in *CCT8* (Figure S2). Consequently, we designated *BnaC01.CCT8* ^*A507V*^ as the candidate gene regulating SL and SW in *ssl*.

The construction of the *pBnaC01.CCT8::GUS* (*β‐glucuronidase*) expression vector and subsequent heterologous expression in *Arabidopsis* revealed that *BnaC01.CCT8* was expressed in nearly all organs, including root tips, lateral roots, leaves, stems, flower buds, siliques and seeds (Figure 2d–i). qRT‐PCR analysis of *BnaC01.CCT8* in NIL‐9D290 and NIL‐*ssl* demonstrated elevated expression levels in siliques, buds and seeds, while reduced expression was observed in other organs, consistent with the expression profile available in the *BnIR* database (https://yanglab.hzau.edu.cn/) (Figure S3a). Notably, the expression level of *BnaC01.CCT8* in NIL‐9D290 was significantly higher than that in NIL‐*ssl* for siliques examined at 3, 7 and 14 days after flowering (DAF), as well as for seeds at 14, 21 and 28 DAF, while no significant differences were observed in other tissues (Figure 2j). Subcellular localization studies conducted in tobacco, utilizing a fusion protein of BnaC01.CCT8 and green fluorescent protein (GFP), indicated that BnaC01.CCT8 was predominantly localized in both the nucleus and cytoplasm, with no discernible differences between BnaC01.CCT8 and BnaC01.CCT8^A507V^ (Figure 2k). These findings suggest that *BnaC01.CCT8* likely plays a crucial role in plant growth and development, particularly in the processes of silique and seed formation.

### *BnaC01* *.* *CCT8* positively regulates SL and TSW

**Figure 3:**
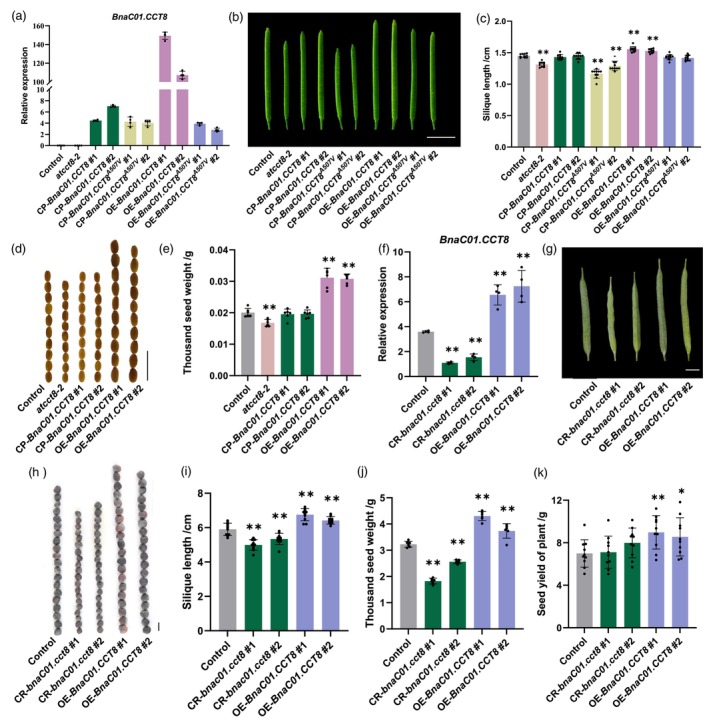
Genetic verification of *BnaC01.CCT8*. (a) qRT‐PCR analysis of *BnaC01.CCT8* in siliques of Arabidopsis, including Col‐0, *atcct8‐2*, heterologous expression lines in *atcct8‐2* (CP*‐BnaC01.CCT8*; CP*‐BnaC01.CCT8* ^*A507V*^) and overexpression lines in Col‐0 (OE*‐BnaC01.CCT8*; OE*‐BnaC01.CCT8* ^*A507V*^). Expression levels were normalized by *AtSAND*. (b) SL phenotypes of lines corresponding to (a). (c) Statistical analysis in (b). (d) Seed phenotypes of Col‐0, *atcct8‐2*, CP*‐BnaC01.CCT8* and OE‐*BnaC01.CCT8*. (e) Statistical analysis in (d). (f) qRT‐PCR analysis of *BnaC01.CCT8* in silique walls from control (Westar), CRISPR/Cas9 lines (CR‐*bnac01.cct8*) and overexpression lines (OE‐*BnaC01.CCT8*). *BnaC02.ACTIN7* was used as a control. (g, h) Phenotype of SL and seed of lines corresponding to (f). (i, j) Statistical analysis in (g) and (h). (k) Statistical analysis of the seed yield of plants in lines corresponding to (f). The #1 and #2 indicate the number of detected positive lines. Scale bar = 0.5 cm in (b), = 2 mm in (d) and (h), = 1 cm in (g). *, *p* < 0.05; **, *p* < 0.01.

The Arabidopsis *cct8‐2* (SALK082168C) mutant exhibited T‐DNA insertions in the 5' untranslated region of *AtCCT8* (Figure S3b), leading to decreased transcript levels as measured by qRT‐PCR (Figure S3c; Xu *et al*., 2011). The *atcct8‐2* mutant displayed shorter SL and lower TSW compared to the control line (Col‐0) (Figure 3b,d). To investigate whether the missense variant in *BnaC01.CCT8* contributes to phenotypic differences, the coding sequences (CDS) of both *BnaC01.CCT8* and *BnaC01.CCT8* ^*A507V*^, driven by the cauliflower mosaic virus (CaMV) 35S promoter, were introduced into the *atcct8‐2* and Col‐0 backgrounds (Figure 3a). Notably, *BnaC01.CCT8* restored the shorter SL of *atcct8‐2* to levels comparable to those of Col‐0, as evidenced by the SL phenotypes of CP*‐BnaC01.CCT8* #1 and #2, which represented heterologous expression lines of *BnaC01.CCT8* in the *atcct8‐2* background (Figure 3b,c). In contrast, CP‐*BnaC01.CCT8* ^*A507V*^ #1 and #2, which expressed *BnaC01.CCT8* ^*A507V*^ in *atcct8‐2*, exhibited shorter SL similar to those of *atcct8‐2* (Figure 3b,c). Additionally, OE*‐BnaC01.CCT8* #1 and #2, which overexpressed *BnaC01.CCT8* in Col‐0, displayed significantly longer SL compared to the control, whereas overexpression of *BnaC01.CCT8* ^*A507V*^ did not affect SL in Col‐0 (Figure 3b,c). Furthermore, *BnaC01.CCT8* restored the SW of *atcct8‐2* to levels observed in Col‐0 (Figure 3d,e). Moreover, the overexpression of *BnaC01.CCT8* resulted in larger seeds in Col‐0 (Figure 3d,e).

To further validate these findings, *BnaC01.CCT8* was overexpressed in the *B. napus* cultivar Westar, with qRT‐PCR confirming the enhanced expression (Figure 3f). Additionally, CRISPR/Cas9 technology was employed to generate two *bnac01.cct8* mutants (CR*‐bnac01.cct8 #1* and *#2*), which exhibited frameshift mutations due to ‐2 bp and +1 bp alterations in the coding sequence, respectively (Figure S3d). The expression levels of *BnaC01.CCT8* were significantly reduced in *bnac01.cct8* mutants (Figure 3f). Notably, both SL and TSW were reduced in the *bnac01.cct8* mutants (CR‐*bnac01.cct8* #1 and #2), while an increase was observed in the *BnaC01.CCT8* overexpression lines (OE‐*BnaC01.CCT8* #1 and #2) when compared to the control (Figure 3g–j). Furthermore, the seed yield per plant was significantly higher in the *BnaC01.CCT8* overexpression lines; however, no significant change was detected in the *bnac01.cct8* mutants relative to the control (Figure 3k). These findings suggest that *BnaC01.CCT8* positively regulates both SL and SW in rapeseed, with the A507 residue being crucial for the molecular functional differentiation of *BnaC01.CCT8*.

### Plant hormone pathways play significant roles in *BnaC01* *.* *CCT8* ‐regulated SL and SW

**Figure 4:**
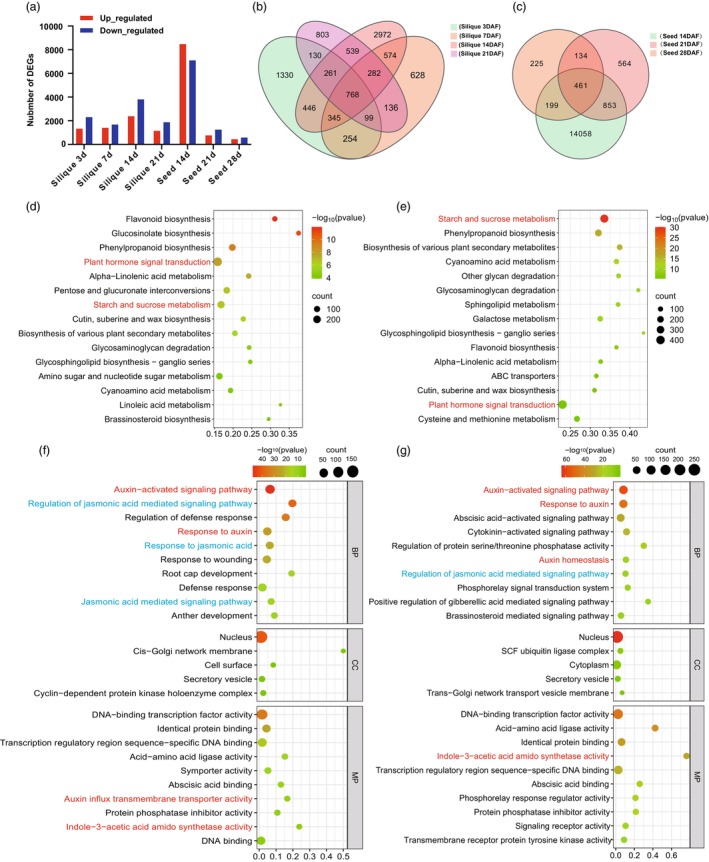
Transcriptome analysis of silique walls and seeds between NIL‐9D290 and NIL‐*ssl*. (a) Statistics of up‐regulated and down‐regulated DEGs in silique walls at 3, 7, 14 and 21 DAF, as well as in seeds at 14, 21 and 28 DAF between NIL‐9D290 and NIL‐*ssl*. (b, c) Venn diagrams of DEGs in silique walls at 3, 7, 14 and 21 DAF (b) and in seeds at 14, 21 and 28 DAF (c). (d, e) KEGG analysis of the top enrichment terms among the total DEGs in silique walls at 3, 7, 14 and 21 DAF (d) and in seeds at 14, 21 and 28 DAF (e). The plant hormone signal transduction pathway and starch and sucrose metabolism are indicated in red font. (f, g) The top GO terms for total DEGs within the plant hormone signal transduction pathway from silique walls at 3, 7, 14 and 21 DAF (f) and in seeds at 14, 21 and 28 DAF (g). Auxin‐related pathways are emphasized in red font and JA‐related pathways are highlighted in blue font. DAF, days after flowering.

To elucidate the role of *BnaC01.CCT8* in silique elongation and seed development, a comparative transcriptome analysis of silique walls at 3, 7, 14 and 21 DAF, as well as seeds at 14, 21 and 28 DAF between NIL‐9D290 and NIL‐*ssl* was conducted. The basic data from the RNA‐seq were presented in Table S2. In silique walls, a total of 9567 differentially expressed genes (DEGs) were identified, with 3633, 3086, 6187 and 3018 DEGs at 3, 7, 14 and 21 DAF, respectively. In seeds, a total of 16 494 DEGs were identified, comprising 15 571, 2021 and 1019 DEGs at 14, 21 and 28 DAF, respectively (Figure 4a–c; Table S3). KEGG (Kyoto Encyclopedia of Genes and Genomes) analysis revealed that the total DEGs in both silique walls and seeds were significantly enriched in plant hormone signal transduction, followed by starch and sucrose metabolism and phenylpropanoid biosynthesis (Figure 4d,e). Furthermore, Gene Ontology (GO) enrichment analysis within the plant hormone signal transduction category indicated that DEGs in silique walls and seeds were highly associated with auxin signalling, including auxin‐activated signalling pathway and response to auxin (Figure 4f,g). Other key enriched pathways in silique walls included the response to JA, JA‐mediated signalling pathway and regulation of JA‐mediated signalling pathway (Figure 4f). In contrast, the DEGs in seeds were significantly enriched in abscisic acid, cytokinin, gibberellin, BR, JA and ethylene signalling pathways (Figure 4g). These results indicate that auxin and JA are predominant in silique development, while multiple hormones play crucial roles in seed development. Moreover, energy metabolism, particularly carbohydrate metabolism, is essential for both silique and seed development in our study (Figure S4a,b).

Silique elongation and seed development are continuous processes. The DEGs across four stages of silique development and three stages of seed development were summarized, leading to the identification of a total of 289 DEGs (Figure S5a). Notably, 82% of these genes were down‐regulated in NIL‐*ssl*, while 18% were up‐regulated (Figure S5b). Among these genes, *BnaA09.CYP86A2*, *BnaC08.GAPC1* (*GLYCERALDEHYDE‐3‐PHOSPHATE DEHYDROGENASE C SUBUNIT 1*) and *BnaA04.ARF17* (*AUXIN‐RESPONSE FACTOR 17*) have been previously reported to be associated with silique and seed development (Huang *et al*., 2022; Li *et al*., 2018; Rius *et al*., 2008). Their differential expression was confirmed by qRT‐PCR (Figure S5c), suggesting that the 289 identified genes may play a crucial role in the processes of silique and seed development.

### *BnaC01* *.* *CCT8* regulates silique and seed development by altering auxin and JA levels in siliques

**Figure 5:**
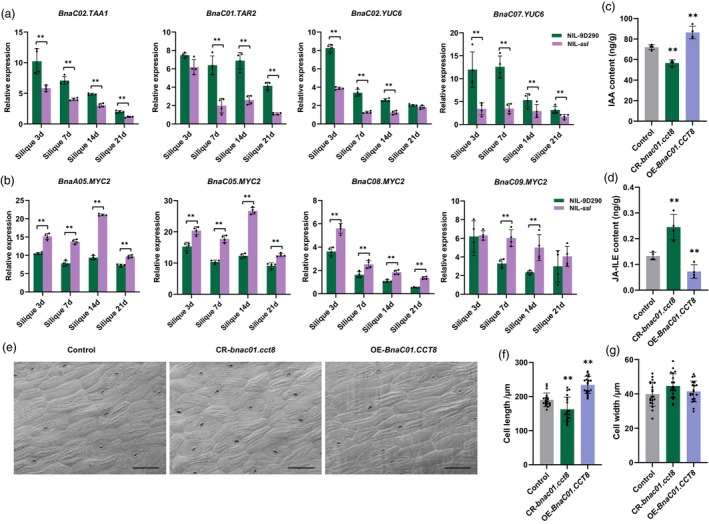
Pathways‐related genes expression and contents of IAA and JA, and cell morphological analysis in silique walls of *BnaC01.CCT8*‐related lines. (a, b) qRT‐qPCR analysis of IAA synthesis genes (a) and JA‐mediated *BnaMYC2* genes (b) in the silique walls of NIL‐9D290 and NIL‐*ssl. BnaC02.ACTIN7* was used as a control. (c, d) Quantification of IAA and JA‐ILE levels in the silique walls of control (Westar), CR‐*bnac01.cct8*, and OE‐*BnaC01.CCT8* lines at 14 DAF. (e) SEM of epidermal cells in silique wall of control (Westar), CR‐*bnac01.cct8*, and OE‐*BnaC01.CCT8* lines at maturity. Scale bar = 100 μm. (f, g) Comparisons of cell length and cell width in silique epidermal cells of (e). **, *p* < 0.01.

The roles of IAA and JA in the regulation of SL have been documented (Shi *et al*., 2019; Zhang *et al*., 2024). Transcriptome analysis revealed that genes associated with IAA synthesis, specifically *TAA1* (*TRYPTOPHAN AMINOTRANSFERASE OF ARABIDOPSIS 1*), *TAR2* (*TRYPTOPHAN AMINOTRANSFERASE RELATED 2*) and *YUC6* (*FLAVIN‐CONTAINING MONOOXYGENASE*), were significantly down‐regulated in the silique walls and seeds of NIL‐*ssl* (Figure S6a). Additionally, qRT‐PCR confirmed that *BnaC02.TAA1*, *BnaC01.TAR2*, *BnaC02.YUC6* and *BnaC07.YUC6* were significantly down‐regulated in the silique walls of NIL‐*ssl*, corroborating the transcriptome results (Figure 5a). Most members of the *EXPANSIN* (*EXPs*) family, known for enhancing cell wall expansion in response to auxin (Majda and Robert, 2018), were significantly down‐regulated in the silique walls of NIL‐*ssl*, particularly at 14 DAF (Figure S6b). In contrast, primary JA biosynthetic genes, including *LOX* (*LIPOXYGENASE*), *AOS* (*ALLENE OXIDE SYNTHASE*), *AOC* (*ALLENE OXIDE CYCLASE*) and *OPR3* (*OXOPHYTODIENOATE‐REDUCTASE 3*), exhibited predominantly higher expression levels in the silique walls of NIL‐*ssl* (Figure S6c). qRT‐PCR analysis demonstrated that members of the *BnaMYC2* family, which function as the principal transcription factors regulating JA‐mediated gene expression (Chen *et al*., 2011), exhibited elevated expression levels in the silique walls of NIL‐*ssl* (Figure 5b).

To further elucidate the role of *BnaC01.CCT8* in the synthesis of IAA and JA, the contents of IAA and JA in OE*‐BnaC01.CCT8*, CR*‐bnac01.cct8* and Westar lines were assessed. As anticipated, compared to the control, the OE‐*BnaC01.CCT8* lines exhibited significantly elevated levels of IAA and reduced levels of JA‐isoleucine (JA‐ILE), the biologically active form of JA. Conversely, the CR*‐bnac01.cct8* lines displayed decreased IAA and increased JA‐ILE (Figure 5c,d). Scanning electron microscopy (SEM) analyses revealed that the epidermal cell length in the silique wall was significantly greater in the OE*‐BnaC01.CCT8* lines, while it was shorter in the CR*‐bnac01.cct8* lines compared to the control. However, no significant differences in cell width were observed (Figure 5e–g). These findings suggest *BnaC01.CCT8* contributes to cell elongation in the silique wall by positively modulating auxin synthesis and negatively regulating JA synthesis in siliques.

### *BnaC01* *.* *CCT8* collaborates with *BnaA09* *.* *ARF18* to regulate silique length

**Figure 6:**
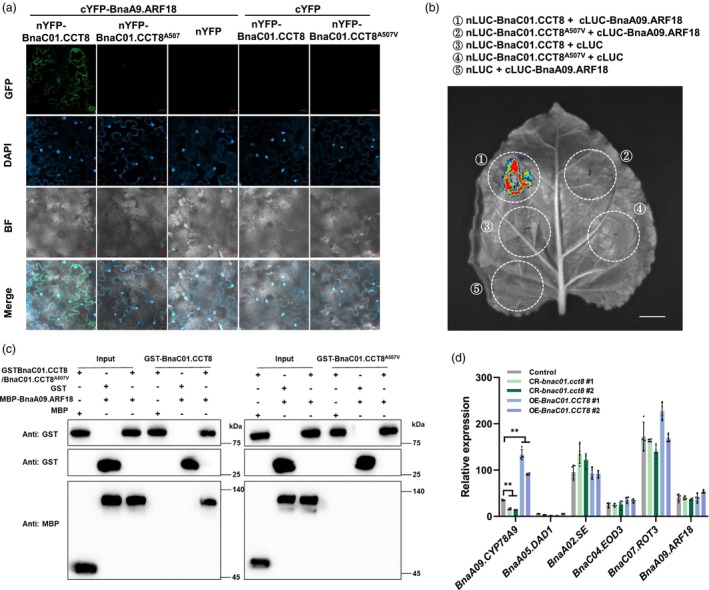
The interaction between BnaC01.CCT8 and BnaA09.ARF18. (a) BiFC assay conducted in *N. benthamiana* leaves using BnaC01.CCT8 or BnaC01.CCT8^A507V^ in conjunction with BnaA09.ARF18. BF, bright field; cYFP/nYFP, C‐terminal/N‐terminal yellow fluorescent protein. DAPI, a nuclear dye. Merge, a combination of GFP signal, bright field and DAPI staining. (b) LCI assay performed in *N. benthamiana* leaves between BnaC01.CCT8, BnaC01.CCT8^A507V^ with BnaA09.ARF18. Five distinct combinations, including three negative controls, were injected into separate areas of the leaf. nLUC/cLUC refer to N‐terminal/C‐terminal luciferase. (c) In vitro pull‐down assay between BnaC01.CCT8, BnaC01.CCT8^A507V^ and BnaA09.ARF18, utilizing GST and MBP. (d) qRT‐PCR analysis of *BnaA09.ARF18*, *BnaC04.EOD3*, *BnaC07.ROT3*, *BnaA05.DAD1* and *BnaA02.SE* in control (Westar), mutation lines (CR*‐bnac01.cct8 #1*and *#2*) and overexpression lines (OE*‐BnaC01.CCT8 #1* and *#2*). *BnaC02.ACTIN7* was used as a control. **, *p* < 0.01. Scale bar = 20 μm in (a), 1 cm in (b).

*BnaA09.ARF18* regulates cell growth in the silique wall through an auxin‐response pathway (Liu *et al*., 2015). Utilizing bimolecular fluorescence complementation assays (BiFC), significant fluorescence signals were observed in the epidermal cells of *Nicotiana benthamiana* leaves co‐expressing nYFP‐BnaC01.CCT8 and cYFP‐BnaA09.ARF18. In contrast, the interaction signal between nYFP‐BnaC01.CCT8^A507V^ and cYFP‐BnaA09.ARF18 was nearly undetectable (Figure 6a). To further investigate these distinct interactions, luciferase complementation imaging (LCI) assays were conducted in *N. benthamiana* leaves. The results indicated that BnaC01.CCT8 interacted with BnaA09.ARF18, as demonstrated by stronger luciferase activity; conversely, the interaction between BnaC01.CCT8^A507V^ and BnaA09.ARF18 exhibited no luciferase activity, consistent with the negative control (Figure 6b). Furthermore, glutathione S‐transferase (GST) pull‐down assays demonstrated that the fusion protein MBP‐BnaA09.ARF18 was successfully pulled down by GST‐BnaC01.CCT8, but not by GST‐BnaC01.CCT8^A507V^ (Figure 6c). These findings indicate that BnaC01.CCT8 interacts with BnaA09.ARF18, while the A507V mutation disrupts this interaction.

In addition, the expression of several known SL‐related genes was detected in NIL‐9D290 and NIL‐*ssl*, including *BnaA09.ARF18*, *BnaA09.CYP78A9*, *BnaC04.EOD3*, *BnaC07.ROT3*, *BnaA05.DAD1* and *BnaA02.SE*. Among these genes, only *BnaA09.CYP78A9* was proved to be significantly down‐regulated in NIL‐*ssl* during silique development by transcriptome and qRT‐PCR analyses (Figure S7a). Furthermore, *BnaA09.CYP78A9* was found to be up‐regulated in OE*‐BnaC01.CCT8* lines and down‐regulated in CR*‐bnac01.cct8* lines; however, the other genes did not display significant changes (Figure 6d). Notably, transcriptomic analysis revealed that the overexpression of *BnaA09.CYP78A9* did not influence the expression of *BnaC01.CCT8* in siliques (Shi *et al*., 2019; Ye *et al*., 2023). Additionally, *BnaC01.CCT8* was significantly up‐regulated in the overexpression lines of *BnaA02.SE* (Figure S7b) (Zhang *et al*., 2024). These results suggest that *BnaC01.CCT8* may function downstream of *BnaA02.SE* and upstream of *BnaA09.CYP78A9*, thereby positively regulating auxin signalling and influencing silique development.

### Analysis of excellent *BnaC01* *.* *CCT8* haplotypes in germplasm resources

**Figure 7:**
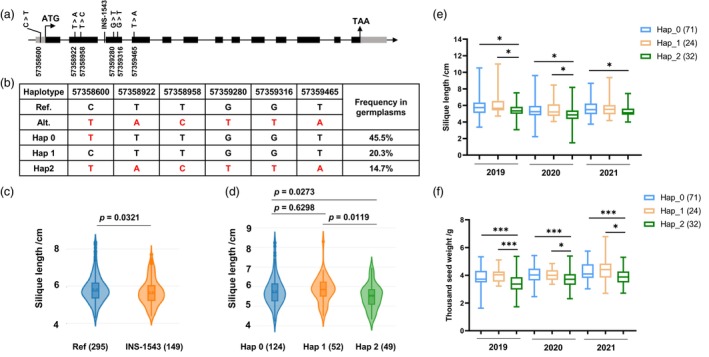
Haplotype analysis of *BnaC01.CCT8* in natural germplasms. (a) The location of SV and SNP variations in the structure of *BnaC01.CCT8*. The locations of variations are indicated below the gene structure. (b) Three main haplotypes of *BnaC01.CCT8* and their distribution frequencies in natural germplasm, with data sourced from *BnVIR*. (c) SL comparison between two haplotypes with SV differences. (d) SL comparison between three main haplotypes with SNP differences. (e, f) SL (e) and TSW (f) of *BnaC01.CCT8* haplotypes in 127 inbred accessions of *B. napus*. 2019, 2020 and 2021 represent the SL and TSW data collected from 3 years, respectively. The number in parentheses indicate the number of lines corresponding to this haplotype. *, *p* < 0.05; ***, *p* < 0.001.

Using the *Brassica napus Variation Information Resource* (*BnVIR*) (Yang *et al*., 2022), a structural variation (SV) and 81 SNPs were identified within the *BnaC01.CCT8* gene. These 81 SNPs were distributed across introns, exons and both the 5′ and 3' UTRs (Figure S8a; Table S4). However, the SNP‐57361321 in *ssl* was not detected in the rapeseed germplasm resources. Phenotypic analysis revealed that the SV, characterized by a 1543 bp insertion (INS‐1543), was associated with significantly shorter SL (Figure 7c), suggesting a potential loss of function of *BnaC01.CCT8*. Haplotype analysis based on the 81 SNPs identified three primary haplotypes. In comparison to Haplotype 1 (ZS11), Haplotype 0 exhibited a mutation from C to T at 57 358 600 bp on chromosome C01. Haplotype 2 contained six SNPs located at 57 358 600 bp, 57 358 922 bp, 57 358 958 bp, 57 359 280 bp, 57 359 316 bp and 57 359 465 bp on chromosome C01 (Figure 7a,b). Among 1163 rapeseed germplasms, the proportions of Haplotype 0, Haplotype 1 and Haplotype 2 were 45.5%, 20.3% and 14.7%, respectively (Figure 7b). In germplasm resources with SL phenotypic data, Haplotype 2 was classified as an unfavourable genotype due to its significantly shorter SL compared to Haplotype 0 and Haplotype 1 (Figure 7d).

To validate these findings, the SV and haplotypes were analysed in 127 inbred accessions of *B. napus*, correlating SL and TSW data over 3 years. Among these accessions, Haplotype 2 consistently exhibited shorter SL and smaller TSW across the 3 years, while no significant differences were observed between Haplotype 0 and Haplotype 1 (Figure 7e,f). However, INS‐1543 of *BnaC01.CCT8* were not detected in these accessions (Figure S9a,b). Furthermore, haplotype analysis of the homologues of *BnaC01.CCT8* (*BnaA01G0405500ZS*, *BnaA03G0293100ZS*, *BnaC03G0351700ZS*) in *BnVIR* revealed no differences in SL and TSW among the primary haplotypes (Figure S8b–g). Based on sequence and phenotypic variation, Haplotype 0 and Haplotype 1 can be integrated into a superior haplotype of *BnaC01.CCT8* for the genetic improvement of high SL and SW.

## Discussion

### *BnaC01* *.* *CCT8* is a novel gene that positively regulates both SL and SW

SL and SW are quantitative traits typically governed by polygenic inheritance and exhibit high heritability (Chen *et al*., 2007; Yang *et al*., 2012; Zhang *et al*., 2011). The complexity of the allopolyploid genome, along with the influence of various environmental factors on SL and SW, presents significant challenges for the cloning of QTL in rapeseed. In this study, *BnaC01.CCT8* was successfully identified through a map‐based cloning approach using a F_2:5_ population derived from the *ssl* mutant. Notably, this study demonstrated that *BnaC01.CCT8* positively regulates both SL and SW in *Arabidopsis* and *B. napus* (Figure 3). Numerous pleiotropic QTL associated with SL and SW have been identified in rapeseed (Hu *et al*., 2022; Liu *et al*., 2015; Raboanatahiry *et al*., 2018; Shi *et al*., 2019; Zhang *et al*., 2024; Zhou *et al*., 2022); however, the *BnaC01.CCT8* locus is absent from the reported loci. Furthermore, since *AtCCT8* regulates stem cell development, *atcct8* mutations in *Arabidopsis* typically result in abnormal growth throughout development (Xu *et al*., 2011). To date, the regulation of SL and SW by *CCT8* has not been reported. These findings suggest that *BnaC01.CCT8* functions as a novel gene that positively regulates both SL and SW.

### *BnaC01* *.* *CCT8* positively regulates SL and SW primarily through auxin and JA signalling

Numerous studies have demonstrated that various hormones, including auxin, abscisic acid (ABA), BR, cytokinin (CTK), ethylene (ETH), gibberellin (GA) and JA, play crucial roles in regulating silique and seed development in rapeseed (Hussain *et al*., 2022; Liu *et al*., 2015; Shi *et al*., 2019; Zhang *et al*., 2024). The DEGs in silique walls between NIL‐9D290 and NIL‐*ssl* were significantly enriched in auxin and JA signalling pathways (Figure 4f). Interestingly, the overexpression of *BnaA02.SE* increased IAA content and reduced JA content, while increasing the expression of *BnaC01.CCT8* (Figure S7b) (Zhang *et al*., 2024). In contrast, the expression of *BnaA02.SE* was largely unaffected in the NIL‐*ssl*, CR*‐bnac01.cct8* and OE*‐BnaC01.CCT8* lines (Figure 6d and S7a), suggesting that *BnaA02.SE* may function upstream of *BnaC01.CCT8*. *BnaA09.CYP78A9* has frequently been reported to positively regulate SL and SW by influencing auxin content (Li *et al*., 2018; Shen *et al*., 2019; Shi *et al*., 2019; Ye *et al*., 2023). Our findings indicated that *BnaA09.CYP78A9* was down‐regulated in the NIL‐*ssl* and CR*‐bnac01.cct8* lines, while it was up‐regulated in the OE*‐BnaC01.CCT8* lines (Figures 6d and S7a). Notably, overexpression of *BnaA09.CYP78A9* did not alter the expression of *BnaC01.CCT8* (Shi *et al*., 2019; Ye *et al*., 2023), suggesting that *BnaA09.CYP78A9* may function downstream of *BnaC01.CCT8*. Furthermore, BnaA09.ARF18, an auxin‐responsive factor, interacted with BnaC01.CCT8; however, the A507V mutation in BnaC01.CCT8 severely disrupted this interaction (Figure 6a–c), potentially leading to altered auxin responses in siliques.

**Figure 8:**
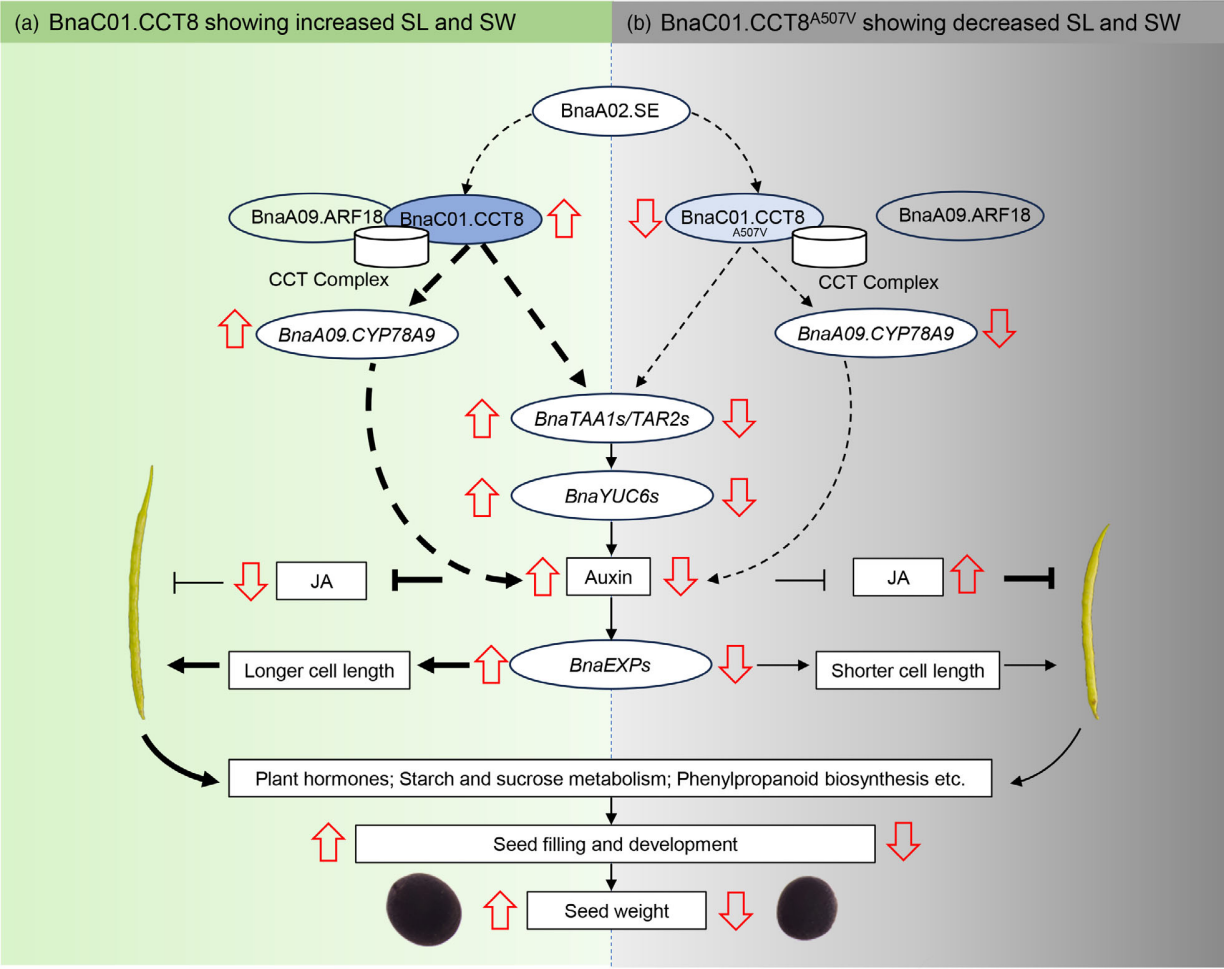
The proposed working module of BnaC01.CCT8 in regulating silique and seed development in rapeseed. (a) The working model of BnaC01.CCT8, which interacts with BnaA09.ARF18 to promote the accumulation of IAA, inhibit the synthesis of JA and positively regulate SL and SW. (b) The working model of BnaC01.CCT8^A507V^, which leads to decreased IAA and increased JA, and negatively regulates SL and SW. Dashed arrows denote putative regulatory pathways, while solid black arrows signify established regulatory pathways. Bold lines indicate enhanced effects.

Notably, auxin synthesis‐related enzymes such as *BnaTAA1/TAR2s* and *BnaYUC6s* were significantly down‐regulated in the NIL‐*ssl* (Figures 5a and S6a). Elevated auxin levels have been shown to negatively influence JA biosynthesis (Cecchetti *et al*., 2013) and enhance cell wall extensibility through the upregulation of *EXP* family members (Majda and Robert, 2018). In our study, overexpression of *BnaC01.CCT8* exhibited significantly higher IAA and lower JA levels (Figure 5c,d), which may alleviate the inhibitory effects of JA, thereby increasing SL. Moreover, several *BnaEXPs* were down‐regulated in NIL‐*ssl* (Figure 6Sb). In contrast, in the CR*‐bnac01.cct8* lines, a reduction in IAA content led to an increase in JA and JA‐ILE synthesis (Figure 5c,d), which activated the expression of *BnaMYC2s*, inhibited cell elongation and resulted in shorter SL. Consequently, we proposed a model to elucidate the mechanism by which *BnaC01.CCT8* is involved in auxin signalling and JA signalling in the regulation of SL and SW (Figure 8). Importantly, BnaC01.CCT8 is a typical molecular chaperone, and further investigation is warranted to clarify the molecular mechanisms through which BnaC01.CCT8 participates in auxin signalling.

### Mutation of BnaC01.CCT8 May destroy CCT complex function

The CCT complex plays a crucial role in facilitating the proper folding of tubulins, mitigating protein aggregation during proteotoxic stress, and mediating the intercellular trafficking of mRNA, tobamoviral components and ribonucleoprotein complexes (Ahn *et al*., 2019; Fichtenbauer *et al*., 2012; Llamas *et al*., 2021; Wang *et al*., 2023; Xu *et al*., 2011). The involvement of the CCT complex in intercellular trafficking likely entails the refolding of transported polypeptides, ensuring their functionality upon delivery to adjacent cells (Fichtenbauer *et al*., 2012). Notably, the loss of a single CCT subunit is sufficient to disrupt the assembly and functionality of the entire CCT complex. The mutation from alanine to valine (A507V) occurs within the predicted α‐helix structure of BnaC01.CCT8 (Figure 2c), which may be involved in protein–protein or protein‐nucleic acid interactions, resulting in impaired interaction of BnaC01.CCT8^A507V^ with BnaA09.ARF18 in both the cytoplasm and nucleus (Figure 6a). It has been reported that in tissues exhibiting high responsiveness to auxin, ARF proteins localize to the nucleus, where they either activate or inhibit auxin signalling (Powers *et al*., 2019). Disruption of ARF assembly formation leads to morphological defects and alters transcriptional responsiveness to auxin (Powers *et al*., 2019). Therefore, it would be valuable to explore whether the CCT complex is involved in the folding, assembly and transport of ARF transcription factors, thereby influencing auxin signalling.

### *BnaC01* *.* *CCT8* contributes to genetic improvement of rapeseed yield

Molecular breeding has played an important role in improving crop yield and quality (Wang *et al*., 2024; Zheng *et al*., 2022). However, only a limited number of genes in rapeseed can be directly utilized for the genetic improvement of yield (Liu *et al*., 2015; Shi *et al*., 2019; Tian *et al*., 2023; Zhang *et al*., 2024; Zhou *et al*., 2022). The gene *BnaC01.CCT8* positively regulates SL and SW. The SNP‐57361321 (C to T) in *BnaC01.CCT8* is a mutation caused by EMS mutagenesis, which restricts its applicability in rapeseed germplasm resources. Through haplotype analysis in *BnVIR*, three primary haplotypes based on SNPs and one SV difference were identified for *BnaC01.CCT8*. Phenotypic data indicated that an insertion of 1543 bp (INS‐1543) was significantly associated with a reduction in SL in germplasms (Figure 7c). However, INS‐1543 was not detected in the resequencing data from our germplasm resources, nor through PCR identification of this SV (Figure S9a,b), suggesting that different germplasm populations may exhibit distinct population structures. Furthermore, among the three primary haplotypes, Haplotype 2 was identified as an unfavourable genotype for shorter SL compared to Haplotype 0 and Haplotype 1 (Figure 7d–f); however, there was no significant difference in seed yield per plant (Figure S10). Moreover, the *bnac01.cct8* mutants (CR‐*bnac01.cct8* #1 and #2) showed a substantial reduction in SW and SW per silique (Figures 3i and S11a), while showing an increase in primary branch number and siliques per plant relative to the control (Figure S11b,c). These compensatory morphological changes resulted in no net difference in total seed yield per plant (Figure 3k). This also indicated that the knockout of *BnaC01.CCT8* has led to alterations in energy distribution between the siliques and the branches. The regulatory role of *BnaC01.CCT8* in determining primary branch number suggested its potential as a promising target for optimizing plant architecture in high‐density cultivation systems. In contrast, the *BnaC01.CCT8* overexpression lines (OE‐*BnaC01.CCT8* #1 and #2) exhibited significantly greater SW and SW per silique (Figures 3i and S11a), resulting in a corresponding increase in seed yield per plant (Figure 3k). Collectively, these results demonstrated that upregulating the expression of *BnaC01.CCT8* is an effective strategy for enhancing rapeseed productivity. Our study provides valuable genetic resources and theoretical support for the genetic improvement of rapeseed yield.

## Materials and methods

### Plant materials and trait evaluation

The *ssl* mutant was derived from the inbred line D11 through EMS mutagenesis, following modified protocols (Li *et al*., 2015). Phenotypic observations of *ssl* were conducted during the M_4_ generation. A total of 127 rapeseed germplasm accessions, including 9D290, from a *B. napus* association population (Li *et al*., 2021) were used for haplotype analysis. All rapeseed materials were cultivated at the experimental farm of the Oil Crops Research Institute, Chinese Academy of Agricultural Sciences, in Wuhan, China. The Arabidopsis mutant *atcct8‐2* (SALK_082168C) was obtained from *AraShare* (https://www.arashare.cn/index/). The Arabidopsis were grown in growth chambers under long‐day conditions (16 h of light/8 h of dark). At maturity, pen‐pollinated seeds and siliques from the main inflorescence were collected. The mean length of the 10 longest, well‐developed siliques (excluding the beak) from the main inflorescence was estimated in *B. napus* and *Arabidopsis*.

### Genetic mapping and candidate gene analysis

The line 120, developed from the F_2:5_ population resulting from the cross between *ssl* and 9D290, was utilized for BSA sequencing. Thirty plants exhibiting extreme long and short SL phenotypes were selected to create a longer SL pool and a shorter SL pool, respectively. The pools, along with *ssl*, D11 and 9D290 were resequenced by Beijing Glbizzia Biotechnology Co.,Ltd. The *de novo* assembled genome of ‘ZS11v0’ served as the reference for calculating the Δ(SNP index) (Song *et al*., 2020). A significant locus was considered potentially linked to *ssl* based on a screening threshold corresponding to a confidence level of 99%. The F_2:6_ population was derived from the heterozygous line 120 within the candidate interval for fine mapping. Homozygous SNP loci between *ssl* and 9D290 were developed into penta‐primer amplification refractory mutation system (PARMS) markers for screening recombinant plants within the RIL_2:6_ population by Gentides Biotech (Wuhan, China). The cDNA and genomic sequences of *BanC01.CCT8* were amplified from *ssl*, D11 and 9D290. Three‐dimensional modelling analyses of BnaC01.CCT8 and BnaC01.CCT8^A507V^ were conducted using *ESMFold* (www.biorpa.com). The primer sequences used in this study were all listed in Table S1.

### Reverse‐transcription PCR and qRT‐PCR analysis

NIL‐9D290 and NIL‐*ssl* were obtained from the F_2:5_ population. Total RNA was extracted from various tissues of NIL‐*ssl* and NIL‐9D290 using the RNAprep Pure Plant Kit (DP432, TIANGEN). Following extraction, reverse transcription was performed using the PrimeScript™ RT reagent Kit with gDNA Eraser (RR047A, Takara), after which quantitative PCR was conducted with the ChamQ Universal SYBR qPCR Master Mix (Q711‐02, Vazyme). Gene‐specific primers were employed to detect the mRNA transcripts of the gene under investigation, while the expression of *BnaACTIN7* (*BnaC02G0037200ZS*) served as a reference. Total RNA was extracted from the siliques of wild type, *atcct8‐2* and transgenic lines in *Arabidopsis*, with expression levels normalized to that of *SAND* (*AT2G28390*). The primer sequences used for qRT‐PCR were listed in Table S1.

### Histochemical staining of GUS activity

The 1.8 kb upstream sequence from the initiation codon of *BanC01.CCT8* was amplified and cloned upstream of the GUS reporter gene within the pCAMBIA1300 expression vector. The resulting *pBnaC01.CCT8::GUS* plasmid was introduced into *Agrobacterium tumefaciens* strain GV3101. Arabidopsis (Col‐0) was transformed using the *Agrobacterium*‐mediated floral dip method (Clough and Bent, 1998). Various tissues from Arabidopsis T_3_ transgenic lines were collected and subjected to GUS staining using the GUS Staining Kit (SL7160, Coolabe, Beijing, China). Images of the GUS staining were captured with a Lighttools camera (SONY SENSOR).

### Subcellular localization and bimolecular fluorescence complementation (BiFC)

The CDSs of *BnaC01.CCT8* and *BnaC01.CCT8* ^*A507V*^ were driven by the CaMV 35S promoter in pCAMBIA2300 vectors, incorporating a GFP tag at the C‐terminus for subcellular localization. In BiFC assays, the CDSs of *BnaC01.CCT8* and *BnaC01.CCT8* ^*A507V*^ were inserted into the N‐terminal fusion yellow fluorescent protein (YFP) vector pXY106, resulting in the constructs BnaC01.CCT8‐nYFP and BnaC01.CCT8^A507V^‐nYFP, respectively. Additionally, the CDS of *BnaA09.ARF18* was cloned into the C‐terminal fusion YFP vector pXY104 to produce BnaA09.ARF18‐cYFP. The resulting plasmids were transformed into *Agrobacterium* strain GV3101 and introduced into *N. benthamiana* leaves via syringe injection for transient expression (Sparkes *et al*., 2006). Two days post‐injection, GFP fluorescence was detected using a Leica SP8 laser scanning confocal microscope, with an excitation wavelength of 488 nm and an emission wavelength ranging from 500 to 550 nm. Nuclei were visualized using 4', 6‐diamidino‐2‐phenylindole (DAPI) staining at a concentration of 10 μg/mL for 10 min.

### Overexpression and knockout vector constructs

The CDSs of *BnaC01.CCT8* and *BnaC01.CCT8* ^*A507V*^ were driven by the CaMV 35S promoter in pCAMBIA2300 vectors to create overexpression lines. For the gene knockout constructs, single guide RNAs (sgRNAs) were designed with the CRISPR2 tool (http://crispr.hzau.edu.cn/CRISPR2/) (Liu *et al*., 2017), and the target sequences were subsequently amplified and cloned into the BGK01 vector (BI0GLE GeneTech). The resulting plasmids for both overexpression and knockout were introduced into the *Agrobacterium* strain GV3101 and transformed into either *Arabidopsis* (Col‐0), *atcct8‐2*, or the *B. napus* cultivar (Westar). For each homozygous T_3_ transgenic line, 10 individual plants were used to measure SL, while six individual plants were utilized for determining TSW.

### RNA‐seq data analysis

Silique walls were harvested at 3, 7, 14 and 21 DAF, along with seeds collected at 14, 21 and 28 DAF from NIL‐*ssl* and NIL‐9D290 for transcriptome analysis. Three biological replicates were performed for each sample. Total RNA was extracted, and transcriptomes were sequenced using the DNBSEQ platform (Shenzhen Huada Genomics Co., China). HISAT software was employed to align clean reads to the reference genome ‘ZS11v0’ (Song *et al*., 2020). Gene expression levels were quantified in Transcripts Per Million (TPM) to account for variations in gene lengths and sequencing discrepancies. DEGs were identified based on a *q*‐value ≤0.05 and a log2 fold change ≥1. KEGG and GO analyses were conducted using an online server (biosys.bgi.com), and the results were visualized using an online platform for data analysis (https://www.bioinformatics.com.cn). A heatmap illustrating mRNA expression levels was generated using TBtools (Chen *et al*., 2020).

### Quantification of IAA and JA‐ILE in *B. napus*

Fresh silique samples were collected at 14 DAF from control, OE*‐BnaC01.CCT8*, and CR‐*bnac01.cct8* lines. The IAA and JA‐ILE content was analysed by Metware (Wuhan, China) using the AB Sciex QTRAP 6500 LC–MS/MS platform with four biological replicates conducted for each sample.

### Scanning electron microscopy

Silique valves were harvested from mature siliques of the control, OE*‐BnaC01.CCT8* lines and CR‐*bnac01.cct8* lines, with three biological replicates conducted for each sample. The harvested valves were subsequently fixed in 2.5% glutaraldehyde in a 0.1 M phosphate buffer. Following fixation, the samples underwent dehydration through a graded ethanol series, were dried using a critical point dryer, and were then sputter‐coated with gold using Nanotech SEMPrep II sputter coater. Imaging was performed using a JSM‐6390 scanning electron microscope. Cell length and width within the silique were measured using ImageJ software.

### Luciferase complementation imaging (LCI) assay

In LCI assays, CDSs of *BnaC01.CCT8* and *BnaC01.CCT8* ^*A507V*^ were cloned into the pCMBIA1300‐nLUC vector, while the CDS of *BnaA09.ARF18* was cloned into the pCMBIA1300‐cLUC vector. These vectors were subsequently transformed into the *Agrobacterium tumefaciens* strain GV3101 and injected into *N. benthamiana* leaves using a syringe for transient expression (Kim *et al*., 2017; Wang *et al*., 2022). To detect luciferase, 1 mM D‐luciferin was applied to the *N. benthamiana* leaves, followed by a 5 min incubation in the dark to quench fluorescence 48 h post‐injection. LCI images were captured using a low‐light cooled charge‐coupled device (CCD) imaging system (Night OWL II LB 983, Berthold).

### In vitro pull‐down assay

Recombinant proteins GST‐BnaC01.CCT8, GST‐BnaC01.CCT8^A507V^ and MBP‐BnaA09.ARF18 were purified from *E. coli*. The proteins GST‐BnaC01.CCT8, GST‐BnaC01.CCT8^A507V^ or GST were incubated with glutathione beads at 4 °C for 2 h, followed by a 1 h incubation with MBP‐BnaA09.ARF18. After elution from the beads, the proteins were subjected to immunoblot analysis using an anti‐MBP antibody to detect MBP‐BnaA09.ARF18.

### Haplotype analysis

Haplotype analysis and SV analyses were conducted using a single‐locus model on *BnVIR* (https://yanglab.hzau.edu.cn/BnVIR). Phenotypic and SNP data were employed to examine the relationship between phenotypic traits and genetic variation (Liu *et al*., 2016). Haplotypes with a frequency exceeding 5% in germplasm resources were considered to be primary haplotypes. To verify the presence of superior haplotypes, phenotypic and genotypic data were collected from 127 inbred lines (Li *et al*., 2021). The SL and TSW data were gathered over the 2018–2019, 2019–2020 and 2020–2021 growing seasons. For each accession, eight individuals were assessed for phenotype, and the mean of three replicates was calculated.

### Statistical analysis

Statistical analyses and chart visualizations were performed using GraphPad Prism 9 (GraphPad Software). T‐tests (and nonparametric tests) and multiple t‐tests (and nonparametric tests) were conducted to analyze the significance of differences.

## Conflict of interest

The authors declare that they have no competing interests.

## Author contributions

Hanzhong Wang and Jinxing Tu conceived the project. Xiaoling Dun and Xinfa Wang supervised the project and modified the paper. Zhaoyang Qu, Ze Tian, Liqing Wei, Chao Wang, Lieqiong Kuang, and Jiaqi Yan performed the experiments. Nian Wang performed the haplotype analysis. Zhaoyang Qu analysed the data and wrote the paper. Furong Wang corrected the paper. All authors contributed to the article and approved the submitted version.

## Supporting information


**Figure S1** Frequency distribution of silique length in line L120 from F_2:5_ population and agronomic traits of NIL‐9D290 and NIL‐*ssl*.
**Figure S2** Sequence alignment of *CCT8* in *Arabidopsis* and *B. napus*.
**Figure S3** Expression pattern and mutant gene structure of *BnaC01.CCT8* and *atcct8‐2*.
**Figure S4** GO analysis of starch and sucrose metabolism.
**Figure S5** Core DEGs in silique and seed development.
**Figure S6** The expression heatmaps for auxin biosynthesis genes, *BnaEXP* genes and JA biosynthesis genes.
**Figure S7** The expression of several known SL‐related genes in silique development.
**Figure S8** Analysis of variation information and haplotype of homologous genes of *BnaC01.CCT8*.
**Figure S9** PCR identification of structural variants (SV).
**Figure S10** Haplotype analysis of *BnaC01.CCT8* in seed yield of plants.
**Figure S11** Changes of various yield‐related traits of gene‐edited and overexpressed lines of *BnaC01.CCT8* at maturity in the field.

**Table S1** The primers used in this study.
**Table S2** The basic data of the RNA‐seq analysis.
**Table S3** The fold change and *q*‐value of DEGs when comparing NIL‐*ssl* to NIL‐9D290 in silique walls and seeds at various developmental stages.
**Table S4** The 81 SNPs of *BnaC01.CCT8* in rapeseed germplasm resources based on *BnVIR*.

## Data Availability

The supplementary data of this article are available online. The RNA‐seq data of NIL‐9D290 and NIL‐*ssl* in developing siliques and seeds, as well as the BSA analysis data, have been deposited in the NCBI database under the BioProject accession PRJNA1229156.

## References

[pbi70184-bib-0001] Ahn, H.K. , Yoon, J.T. , Choi, I. , Kim, S. , Lee, H.S. and Pai, H.S. (2019) Functional characterization of chaperonin containing T‐complex polypeptide‐1 and its conserved and novel substrates in *Arabidopsis* . J. Exp. Bot. 70, 2741–2757. 30825377 10.1093/jxb/erz099 PMC6506772

[pbi70184-bib-0002] Bennett, E.J. , Roberts, J.A. and Wagstaff, C. (2011) The role of the pod in seed development: strategies for manipulating yield. New Phytol. 190, 838–853. 21507003 10.1111/j.1469-8137.2011.03714.x

[pbi70184-bib-0003] Cecchetti, V. , Altamura, M.M. , Brunetti, P. , Petrocelli, V. , Falasca, G. , Ljung, K. , Costantino, P. *et al*. (2013) Auxin controls Arabidopsis anther dehiscence by regulating endothecium lignification and jasmonic acid biosynthesis. Plant J. 74, 411–422. 23410518 10.1111/tpj.12130

[pbi70184-bib-0004] Chay, P. and Thurling, N. (2010) Identification of genes controlling pod length in spring rapeseed, *Brassica napus* L., and their utilization for yield improvement. Plant Breed. 103, 54–62.

[pbi70184-bib-0005] Chen, W. , Zhang, Y. , Liu, X. , Chen, B. , Tu, J. and Fu, T. (2007) Detection of QTL for six yield‐related traits in oilseed rape (*Brassica napus*) using DH and immortalized F(2) populations. Theor. Appl. Genet. 115, 849–858. 17665168 10.1007/s00122-007-0613-2

[pbi70184-bib-0006] Chen, Q. , Sun, J. , Zhai, Q. , Zhou, W. , Qi, L. , Xu, L. , Wang, B. *et al*. (2011) The basic helix‐loop‐helix transcription factor MYC2 directly represses *PLETHORA* expression during jasmonate‐mediated modulation of the root stem cell niche in *Arabidopsis* . Plant Cell 23, 3335–3352. 21954460 10.1105/tpc.111.089870 PMC3203420

[pbi70184-bib-0007] Chen, C. , Chen, H. , Zhang, Y. , Thomas, H.R. , Frank, M.H. , He, Y. and Xia, R. (2020) TBtools: an integrative toolkit developed for interactive analyses of big biological data. Mol. Plant 13, 1194–1202. 32585190 10.1016/j.molp.2020.06.009

[pbi70184-bib-0008] Clough, S.J. and Bent, A.F. (1998) Floral dip: a simplified method for *Agrobacterium*‐mediated transformation of *Arabidopsis thaliana* . Plant J. 16, 735–743. 10069079 10.1046/j.1365-313x.1998.00343.x

[pbi70184-bib-0009] Cong, Y. , Schröder, G.F. , Meyer, A.S. , Jakana, J. , Ma, B. , Dougherty, M.T. , Schmid, M.F. *et al*. (2012) Symmetry‐free cryo‐EM structures of the chaperonin TRiC along its ATPase‐driven conformational cycle. EMBO J. 31, 720–730. 22045336 10.1038/emboj.2011.366 PMC3273382

[pbi70184-bib-0010] Dekker, C. , Stirling, P.C. , McCormack, E.A. , Filmore, H. , Paul, A. , Brost, R.L. , Costanzo, M. *et al*. (2008) The interaction network of the chaperonin CCT. EMBO J. 27, 1827–1839. 18511909 10.1038/emboj.2008.108 PMC2486426

[pbi70184-bib-0011] Fichtenbauer, D. , Xu, X.M. , Jackson, D. and Kragler, F. (2012) The chaperonin CCT8 facilitates spread of tobamovirus infection. Plant Signal. Behav. 7, 318–321. 22476462 10.4161/psb.19152 PMC3443910

[pbi70184-bib-0012] Freund, A. , Zhong, F.L. , Venteicher, A.S. , Meng, Z. , Veenstra, T.D. , Frydman, J. and Artandi, S.E. (2014) Proteostatic control of telomerase function through TRiC‐mediated folding of TCAB1. Cell 159, 1389–1403. 25467444 10.1016/j.cell.2014.10.059 PMC4329143

[pbi70184-bib-0013] Grantham, J. (2020) The molecular chaperone CCT/TRiC: an essential component of proteostasis and a potential modulator of protein aggregation. Front. Genet. 11, 172. 32265978 10.3389/fgene.2020.00172 PMC7096549

[pbi70184-bib-0014] Gu, J. , Chen, J. , Xia, J. and Hong, D. (2023) *BnaUBP15s* positively regulates seed size and seed weight in *Brassica napus* . Oil Crop Sci. 8, 149–155.

[pbi70184-bib-0015] Horwich, A.L. , Fenton, W.A. , Chapman, E. and Farr, G.W. (2007) Two families of chaperonin: physiology and mechanism. Annu. Rev. Cell Dev. Biol. 23, 115–145. 17489689 10.1146/annurev.cellbio.23.090506.123555

[pbi70184-bib-0016] Hu, J. , Chen, B. , Zhao, J. , Zhang, F. , Xie, T. , Xu, K. , Gao, G. *et al*. (2022) Genomic selection and genetic architecture of agronomic traits during modern rapeseed breeding. Nat. Genet. 54, 694–704. 35484301 10.1038/s41588-022-01055-6

[pbi70184-bib-0017] Huang, J. , Zhao, L. , Malik, S. , Gentile, B.R. , Xiong, V. , Arazi, T. , Owen, H.A. *et al*. (2022) Specification of female germline by microRNA orchestrated auxin signaling in *Arabidopsis* . Nat. Commun. 13, 6960. 36379956 10.1038/s41467-022-34723-6 PMC9666636

[pbi70184-bib-0018] Hussain, Q. , Zhan, J. , Liang, H. , Wang, X. , Liu, G. , Shi, J. and Wang, H. (2022) Key genes and mechanisms underlying natural variation of silique length in oilseed rape (*Brassica napus* L.) germplasm. Crop J. 10, 617–626.

[pbi70184-bib-0019] Joachimiak, L.A. , Walzthoeni, T. , Liu, C.W. , Aebersold, R. and Frydman, J. (2014) The structural basis of substrate recognition by the eukaryotic chaperonin TRiC/CCT. Cell 159, 1042–1055. 25416944 10.1016/j.cell.2014.10.042 PMC4298165

[pbi70184-bib-0020] Khan, M.H.U. , Hu, L. , Zhu, M. , Zhai, Y. , Khan, S.U. , Ahmar, S. , Amoo, O. *et al*. (2020) Targeted mutagenesis of *EOD3* gene in *Brassica napus* L. regulates seed production. J. Cell. Physiol. 236, 1996–2007. 32841372 10.1002/jcp.29986

[pbi70184-bib-0021] Kim, S.H. , Kim, H.S. , Bahk, S. , An, J. , Yoo, Y. , Kim, J.Y. and Chung, W.S. (2017) Phosphorylation of the transcriptional repressor MYB15 by mitogen‐activated protein kinase 6 is required for freezing tolerance in *Arabidopsis* . Nucleic Acids Res. 45, 6613–6627. 28510716 10.1093/nar/gkx417 PMC5499865

[pbi70184-bib-0059] Kitagawa, M. , Wu, P. , Balkunde, R. , Cunniff, P. , Jackson . (2022) An RNA exosome subunit mediates cell‐to‐cell trafficking of a homeobox mRNA via plasmodesmata. Science 375, 177–182. 35025667 10.1126/science.abm0840

[pbi70184-bib-0022] Leitner, A. , Joachimiak, L.A. , Bracher, A. , Mönkemeyer, L. , Walzthoeni, T. , Chen, B. , Pechmann, S. *et al*. (2012) The molecular architecture of the eukaryotic chaperonin TRiC/CCT. Structure 20, 814–825. 22503819 10.1016/j.str.2012.03.007 PMC3350567

[pbi70184-bib-0023] Li, H. , Li, J. , Zhao, B. , Wang, J. , Yi, L. , Liu, C. , Wu, J. *et al*. (2015) Generation and characterization of tribenuron‐methyl herbicide‐resistant rapeseed (*Brasscia napus*) for hybrid seed production using chemically induced male sterility. Theor. Appl. Genet. 128, 107–118. 25504538 10.1007/s00122-014-2415-7

[pbi70184-bib-0024] Li, N. , Song, D. , Peng, W. , Zhan, J. , Shi, J. , Wang, X. , Liu, G. *et al*. (2018) Maternal control of seed weight in rapeseed (*Brassica napus* L.): the causal link between the size of pod (mother, source) and seed (offspring, sink). Plant Biotechnol. J. 17, 736–749. 30191657 10.1111/pbi.13011 PMC6419582

[pbi70184-bib-0025] Li, K. , Wang, J. , Kuang, L. , Tian, Z. , Wang, X. , Dun, X. , Tu, J. *et al*. (2021) Genome‐wide association study and transcriptome analysis reveal key genes affecting root growth dynamics in rapeseed. Biotechnol. Biofuels 14, 178. 34507599 10.1186/s13068-021-02032-7 PMC8431925

[pbi70184-bib-0026] Liu, J. , Hua, W. , Yang, H. , Zhan, G. , Li, R. , Deng, L. , Wang, X. *et al*. (2012) The *BnGRF2* gene (*GRF2‐like* gene from *Brassica napus*) enhances seed oil production through regulating cell number and plant photosynthesis. J. Exp. Bot. 63, 3727–3740. 22442419 10.1093/jxb/ers066 PMC3388832

[pbi70184-bib-0027] Liu, J. , Hua, W. , Hu, Z. , Yang, H. , Zhang, L. , Li, R. , Deng, L. *et al*. (2015) Natural variation in *ARF18* gene simultaneously affects seed weight and silique length in polyploid rapeseed. Proc. Natl. Acad. Sci. U. S. A. 112, E5123–E5132. 26324896 10.1073/pnas.1502160112 PMC4577148

[pbi70184-bib-0028] Liu, S. , Fan, C. , Li, J. , Cai, G. , Yang, Q. , Wu, J. , Yi, X. *et al*. (2016) A genome‐wide association study reveals novel elite allelic variations in seed oil content of *Brassica napus* . Theor. Appl. Genet. 129, 1203–1215. 26912143 10.1007/s00122-016-2697-z

[pbi70184-bib-0029] Liu, H. , Ding, Y. , Zhou, Y. , Jin, W. , Xie, K. and Chen, L.L. (2017) CRISPR‐P 2.0: an improved CRISPR‐Cas9 tool for genome editing in plants. Mol. Plant 10, 530–532. 28089950 10.1016/j.molp.2017.01.003

[pbi70184-bib-0030] Liu, M. , Chang, W. , Yu, M. , Fan, Y. , Shang, G. , Xu, Y. , Niu, Y. *et al*. (2021) Overexpression of *DEFECTIVE IN ANTHER DEHISCENCE 1* increases rapeseed silique length through crosstalk between JA and auxin signaling. Ind. Crop Prod. 168, 113576.

[pbi70184-bib-0031] Llamas, E. , Torres‐Montilla, S. , Lee, H.J. , Barja, M.V. , Schlimgen, E. , Dunken, N. , Wagle, P. *et al*. (2021) The intrinsic chaperone network of Arabidopsis stem cells confers protection against proteotoxic stress. Aging Cell 20, e13446. 34327811 10.1111/acel.13446 PMC8373342

[pbi70184-bib-0032] Majda, M. and Robert, S. (2018) The role of auxin in cell wall expansion. Int. J. Mol. Sci. 19, 951. 29565829 10.3390/ijms19040951 PMC5979272

[pbi70184-bib-0033] Miller, C. , Wells, R. , McKenzie, N. , Trick, M. , Ball, J. , Fatihi, A. , Dubreucq, B. *et al*. (2019) Variation in expression of the HECT E3 ligase *UPL3* modulates LEC2 levels, seed size, and crop yields in *Brassica napus* . Plant Cell 31, 2370–2385. 31439805 10.1105/tpc.18.00577 PMC6790077

[pbi70184-bib-0034] Noormohammadi, A. , Khodakarami, A. , Gutierrez‐Garcia, R. , Lee, H.J. , Koyuncu, S. , König, T. , Schindler, C. *et al*. (2016) Somatic increase of CCT8 mimics proteostasis of human pluripotent stem cells and extends C. elegans lifespan. Nat. Commun. 7, 13649. 27892468 10.1038/ncomms13649 PMC5133698

[pbi70184-bib-0035] Powers, S.K. , Holehouse, A.S. , Korasick, D.A. , Schreiber, K.H. , Clark, N.M. , Jing, H. , Emenecker, R. *et al*. (2019) Nucleo‐cytoplasmic Partitioning of ARF proteins controls auxin responses in *Arabidopsis thaliana* . Mol. Cell 76, 177–190. 31421981 10.1016/j.molcel.2019.06.044 PMC6778021

[pbi70184-bib-0036] Qi, L. , Mao, L. , Sun, C. , Pu, Y. , Fu, T. , Ma, C. , Shen, J. *et al*. (2013) Interpreting the genetic basis of silique traits in *Brassica napus* using a joint QTL network. Plant Breed. 133, 52–60.

[pbi70184-bib-0037] Raboanatahiry, N. , Chao, H. , Dalin, H. , Pu, S. , Yan, W. , Yu, L. , Wang, B. *et al*. (2018) QTL alignment for seed yield and yield related traits in *Brassica napus* . Front. Plant Sci. 9, 1127. 30116254 10.3389/fpls.2018.01127 PMC6083399

[pbi70184-bib-0038] Rius, S.P. , Casati, P. , Iglesias, A.A. and Gomez‐Casati, D.F. (2008) Characterization of Arabidopsis lines deficient in GAPC‐1, a cytosolic NAD‐dependent glyceraldehyde‐3‐phosphate dehydrogenase. Plant Physiol. 148, 1655–1667. 18820081 10.1104/pp.108.128769 PMC2577239

[pbi70184-bib-0039] Shen, W. , Qin, P. , Yan, M. , Li, B. , Wu, Z. , Wen, J. , Yi, B. *et al*. (2019) Fine mapping of a silique length‐ and seed weight‐related gene in *Brassica napus* . Theor. Appl. Genet. 132, 2985–2996. 31321475 10.1007/s00122-019-03400-6

[pbi70184-bib-0040] Shi, L. , Song, J. , Guo, C. , Wang, B. , Guan, Z. , Yang, P. , Chen, X. *et al*. (2019) A CACTA‐like transposable element in the upstream region of *BnaA9.CYP78A9* acts as an enhancer to increase silique length and seed weight in rapeseed. Plant J. 98, 524–539. 30664290 10.1111/tpj.14236

[pbi70184-bib-0041] Song, J.M. , Guan, Z. , Hu, J. , Guo, C. , Yang, Z. , Wang, S. , Liu, D. *et al*. (2020) Eight high‐quality genomes reveal pan‐genome architecture and ecotype differentiation of *Brassica napus* . Nat. Plants 6, 34–45. 31932676 10.1038/s41477-019-0577-7 PMC6965005

[pbi70184-bib-0042] Sparkes, I.A. , Runions, J. , Kearns, A. and Hawes, C. (2006) Rapid, transient expression of fluorescent fusion proteins in tobacco plants and generation of stably transformed plants. Nat. Protoc. 1, 2019–2025. 17487191 10.1038/nprot.2006.286

[pbi70184-bib-0043] Spiess, C. , Miller, E.J. , McClellan, A.J. and Frydman, J. (2006) Identification of the TRiC/CCT substrate binding sites uncovers the function of subunit diversity in eukaryotic chaperonins. Mol. Cell 24, 25–37. 17018290 10.1016/j.molcel.2006.09.003 PMC3339573

[pbi70184-bib-0044] Tian, X. , Yu, X. , Wang, Z. , Guo, L. , Tu, J. , Shen, J. , Yi, B. *et al*. (2023) BnaMPK3s promote organ size by interacting with BnaARF2s in *Brassica napus* . Plant Biotechnol. J. 21, 899–901. 36660879 10.1111/pbi.14013 PMC10106852

[pbi70184-bib-0045] Wang, N. , Zhang, B. , Yao, T. , Shen, C. , Wen, T. , Zhang, R. , Li, Y. *et al*. (2022) Re enhances anthocyanin and proanthocyanidin accumulation to produce red foliated cotton and brown fiber. Plant Physiol. 189, 1466–1481. 35289870 10.1093/plphys/kiac118 PMC9237731

[pbi70184-bib-0046] Wang, S. , Duan, X. , Wang, S. , Hao, L. , Zhang, Y. , Xu, C. , Yu, Y. *et al*. (2023) A chaperonin containing T‐complex polypeptide‐1 facilitates the formation of the PbWoxT1‐PbPTB3 ribonucleoprotein complex for long‐distance RNA trafficking in *Pyrus betulaefolia* . New Phytol. 238, 1115–1128. 36751904 10.1111/nph.18789

[pbi70184-bib-0047] Wang, H. , Li, X. , Meng, B. , Fan, Y. , Khan, S.U. , Qian, M. , Zhang, M. *et al*. (2024) Exploring silique number in *Brassica napus* L.: Genetic and molecular advances for improving yield. Plant Biotechnol. J. 22, 1897–1912. 38386569 10.1111/pbi.14309 PMC11182599

[pbi70184-bib-0048] Xu, X.M. , Wang, J. , Xuan, Z. , Goldshmidt, A. , Borrill, P.G.M. , Hariharan, N. , Kim, J. *et al*. (2011) Chaperonins facilitate KNOTTED1 cell‐to‐cell trafficking and stem cell function. Science 333, 1141–1144. 21868675 10.1126/science.1205727

[pbi70184-bib-0049] Yang, P. , Shu, C. , Chen, L. , Xu, J. , Wu, J. and Liu, K. (2012) Identification of a major QTL for silique length and seed weight in oilseed rape (*Brassica napus* L.). Theor. Appl. Genet. 125, 285–296. 22406980 10.1007/s00122-012-1833-7

[pbi70184-bib-0050] Yang, Y. , Zhu, K. , Li, H. , Han, S. , Meng, Q. , Khan, S.U. , Fan, C. *et al*. (2018) Precise editing of *CLAVATA* genes in *Brassica napus* L. regulates multilocular silique development. Plant Biotechnol. J. 16, 1322–1335. 29250878 10.1111/pbi.12872 PMC5999189

[pbi70184-bib-0051] Yang, Z. , Liang, C. , Wei, L. , Wang, S. , Yin, F. , Liu, D. , Guo, L. *et al*. (2022) BnVIR: bridging the genotype‐phenotype gap to accelerate mining of candidate variations for traits in *Brassica napus* . Mol. Plant 15, 779–782. 35144025 10.1016/j.molp.2022.02.002

[pbi70184-bib-0052] Ye, J. , Liang, H. , Zhao, X. , Li, N. , Song, D. , Zhan, J. , Liu, J. *et al*. (2023) A systematic dissection in oilseed rape provides insights into the genetic architecture and molecular mechanism of yield heterosis. Plant Biotechnol. J. 21, 1479–1495. 37170717 10.1111/pbi.14054 PMC10281607

[pbi70184-bib-0053] Yébenes, H. , Mesa, P. , Muñoz, I.G. , Montoya, G. and Valpuesta, J.M. (2011) Chaperonins: two rings for folding. Trends Biochem. Sci. 36, 424–432. 21723731 10.1016/j.tibs.2011.05.003

[pbi70184-bib-0054] Zhang, L. , Yang, G. , Liu, P. , Hong, D. , Li, S. and He, Q. (2011) Genetic and correlation analysis of silique‐traits in *Brassica napus* L. by quantitative trait locus mapping. Theor. Appl. Genet. 122, 21–31. 20686746 10.1007/s00122-010-1419-1

[pbi70184-bib-0055] Zhang, L. , Yang, B. , Li, X. , Chen, S. , Zhang, C. , Xiang, S. , Sun, T. *et al*. (2024) Integrating GWAS, RNA‐Seq and functional analysis revealed that *BnaA02.SE* mediates silique elongation by affecting cell proliferation and expansion in *Brassica napus* . Plant Biotechnol. J. 22, 2907–2920. 38899717 10.1111/pbi.14413 PMC11536457

[pbi70184-bib-0056] Zheng, M. , Terzaghi, W. , Wang, H. and Hua, W. (2022) Integrated strategies for increasing rapeseed yield. Trends Plant Sci. 27, 742–745. 35501261 10.1016/j.tplants.2022.03.008

[pbi70184-bib-0057] Zhou, X. , Zhang, H. , Wang, P. , Liu, Y. , Zhang, X. , Song, Y. , Wang, Z. *et al*. (2022) *BnaC7.ROT3*, the causal gene of *cqSL‐C7*, mediates silique length by affecting cell elongation in *Brassica napus* . J. Exp. Bot. 73, 154–167. 34486674 10.1093/jxb/erab407

[pbi70184-bib-0058] Zhu, X. , Zhang, L. , Kuang, C. , Guo, Y. , Huang, C. , Deng, L. , Sun, X. *et al*. (2018) Important photosynthetic contribution of silique wall to seed yield‐related traits in *Arabidopsis thaliana* . Photosynth. Res. 137, 493–501. 29959749 10.1007/s11120-018-0532-x

