# Supplementary material for: A chaperonin BnaC01.CCT8 contributes to silique length and seed weight by affecting auxin and jasmonic acid signalling in Brassica napus
Source: Plant Biotechnol J. 2025 Jun 18;23(9):3934–48. doi: 10.1111/pbi.70184 (PMC12392976; doi:10.1111/pbi.70184)
Supplement: Supplementary file 1 — Figure S1 Frequency distribution of silique length in line L120 from F2:5 population and agronomic traits of NIL‐9D290 and NIL‐ssl. Figure S2 Sequence alignment of CCT8 in Arabidopsis and B. napus. Figure S3 Expression pattern and mutant gene structure of BnaC01.CCT8 and atcct8‐2. Figure S4 GO analysis of starch and sucrose metabolism. Figure S5 Core DEGs in silique and seed development. Figure S6 The expression heatmaps for auxin biosynthesis genes, BnaEXP genes and JA biosynthesis genes. Figure S7 The expression of several known SL‐related genes in silique development. Figure S8 Analysis of variation information and haplotype of homologous genes of BnaC01.CCT8. Figure S9 PCR identification of structural variants (SV). Figure S10 Haplotype analysis of BnaC01.CCT8 in seed yield of plants. Figure S11 Changes of various yield‐related traits of gene‐edited and overexpressed lines of BnaC01.CCT8 at maturity in the field. [file PBI-23-3934-s001.docx]

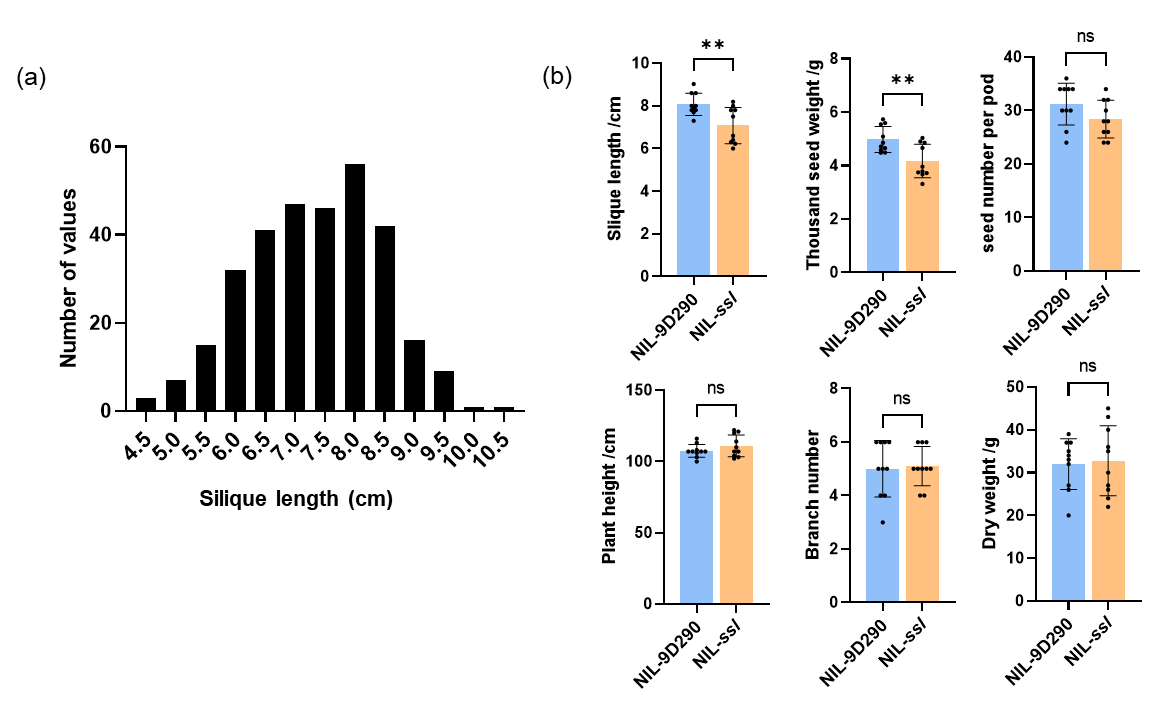


**Supplement figure 1 Frequency distribution of silique length in line L120 from F_2:5_ population and agronomic traits of NIL-9D290 and NIL-*ssl*.**

(a) The frequency distribution of SL at maturity among 316 individuals in L120 line derived from the F_2:5_ population (9D290 × *ssl*). (b) Agronomic traits of NIL-9D290 and NIL-*ssl* at maturity, including silique length, thousand seed weight, seed number per pod, plant height, branch number and dry weight. ns, *p* > 0.05; **, *p* < 0.01.


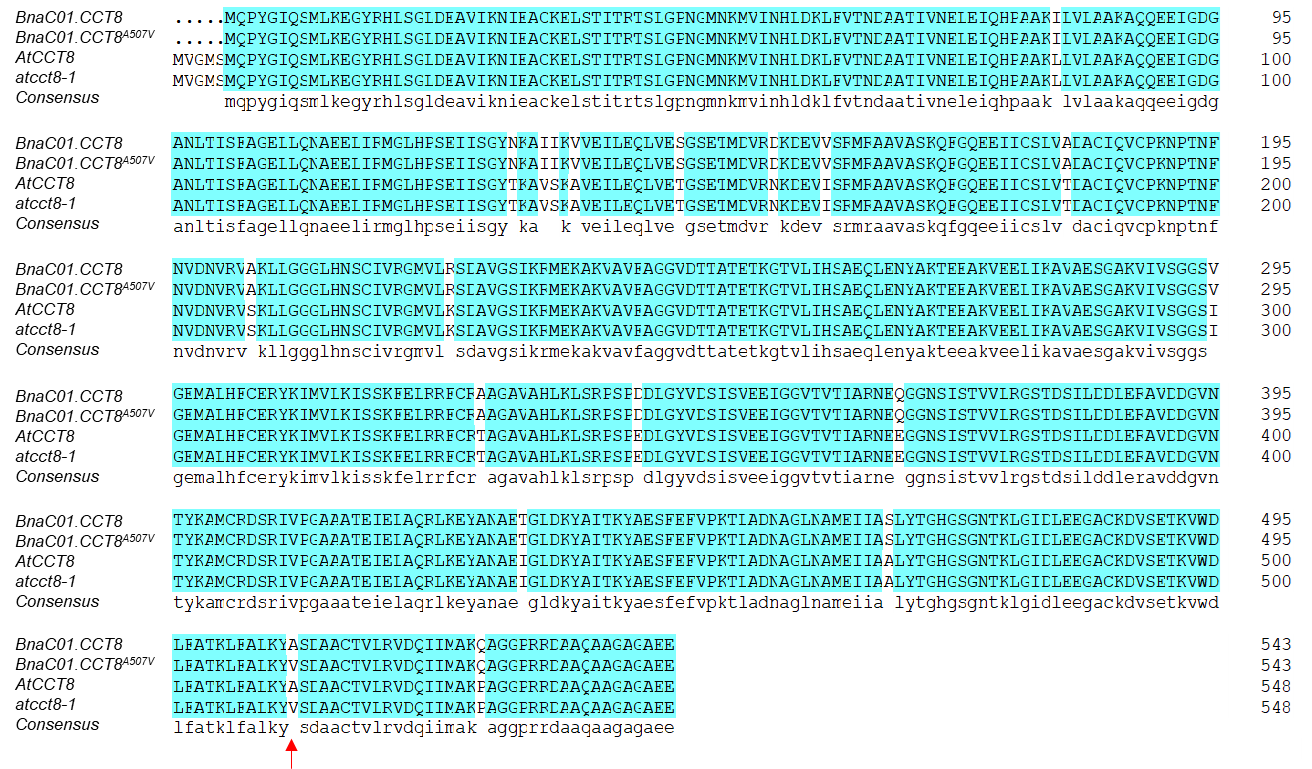


**Supplement figure 2 Sequence alignment of *CCT8* in *Arabidopsis* and *B. napus***.

The multiple sequence alignment of *BnaC01.CCT8*, *BnaC01.CCT8^A507V^*, *AtCCT8* and *atcct8-1* reveals a sequence identity of 96.7% between *AtCCT8* and *BnaC01.CCT8*. The red arrow indicates that *BnaC01.CCT8^A507V^* and *atcct8-1* share the same type of mutation at the identical amino acid site.


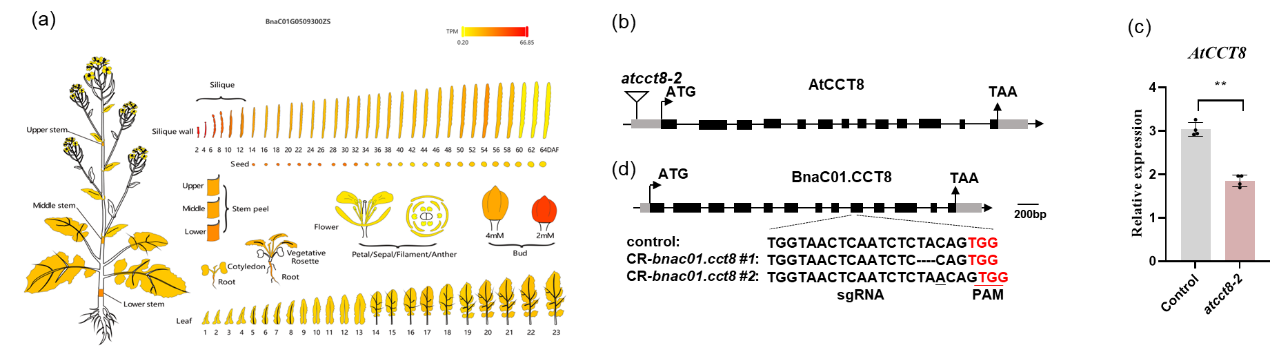


**Supplement figure 3 Expression pattern and mutant gene structure of *BnaC01.CCT8* and *atcct8-2.***

(a) The electronic Fluorescent Pictographic (eFP) images of *BnaC01.CCT8* during the entire growth period of ‘ZS11’ (*https://yanglab.hzau.edu.cn/BnIR/expression_zs11*). The color scale represents transcripts per million (TPM) value. (b) T-DNA insertion in *atcct8-2* is represented as open triangles. (c) qRT-PCR analysis of *AtCCT8* expression in Col-0 and *atcct8-2*. Expression levels were normalized to that of *AtSAND.* **, *p* < 0.01. (d) The gene structures of *BnaC01.CCT8* mutations, designated as CR-*bnac01.cct8* #1 and #2, were generated using the CRISPR/Cas9. The regions targeted by sgRNA are indicated, with the PAM motif sequence emphasized in red. Base deletion is marked by dashes, while base insertion is shown with underlines. Black bars, exons; lines, introns; gray bars, untranslated regions; ATG, start codon; TAA, stop codon.


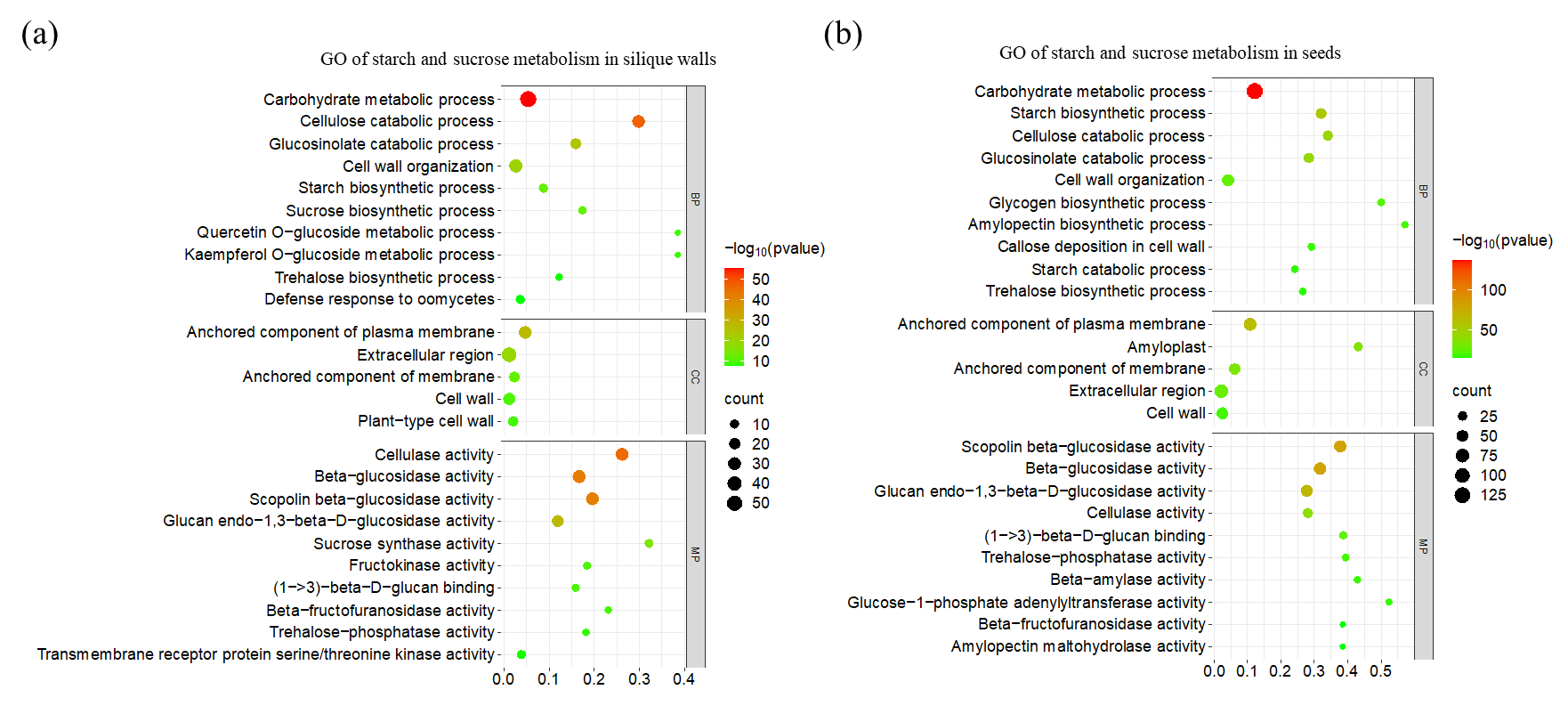


**Supplement figure 4 GO analysis of starch and sucrose metabolism.**

The top GO terms for DEGs in the starch and sucrose metabolism pathway in silique walls (a) and seeds (b).


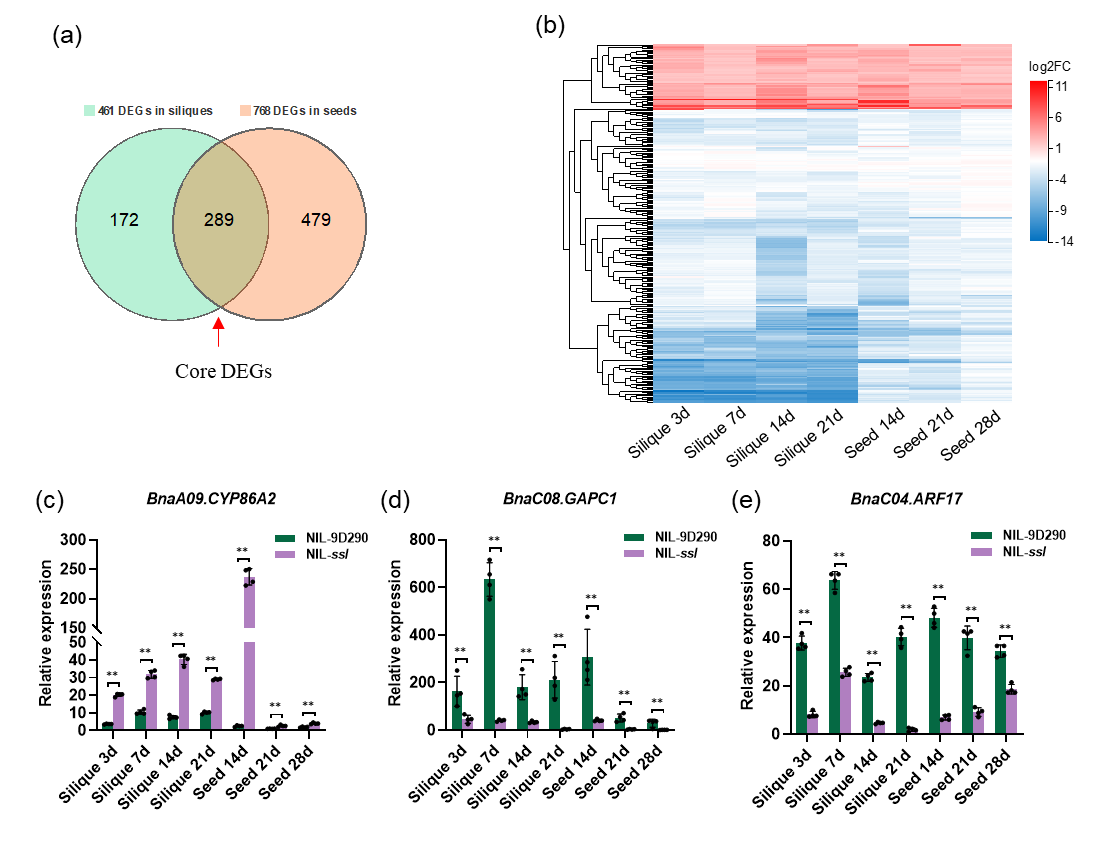


**Supplement figure 5 Core DEGs in silique and seed development.**

(a) Venn diagram illustrates 461 DEGs across four stages of silique development and 768 DEGs across three stages of seed development. The red arrow indicates the 289 core DEGs. (b) Heatmap displays the differential expression of 289 core DEGs, with colors representing the log2 fold change. The 3d, 7d, 14d, 21d, and 28d, represents 3, 7, 14, 21, and 28 days after flowering, respectively. (c-e) qRT-PCR analysis confirms the differential expression of *BnaA09.CYP86A2* (c), *BnaC08.GAPC1* (d), and *BnaC04.ARF17* (e), respectively. *BnaC02.ACTIN7* was used as a control*.* **, *p* < 0.01.


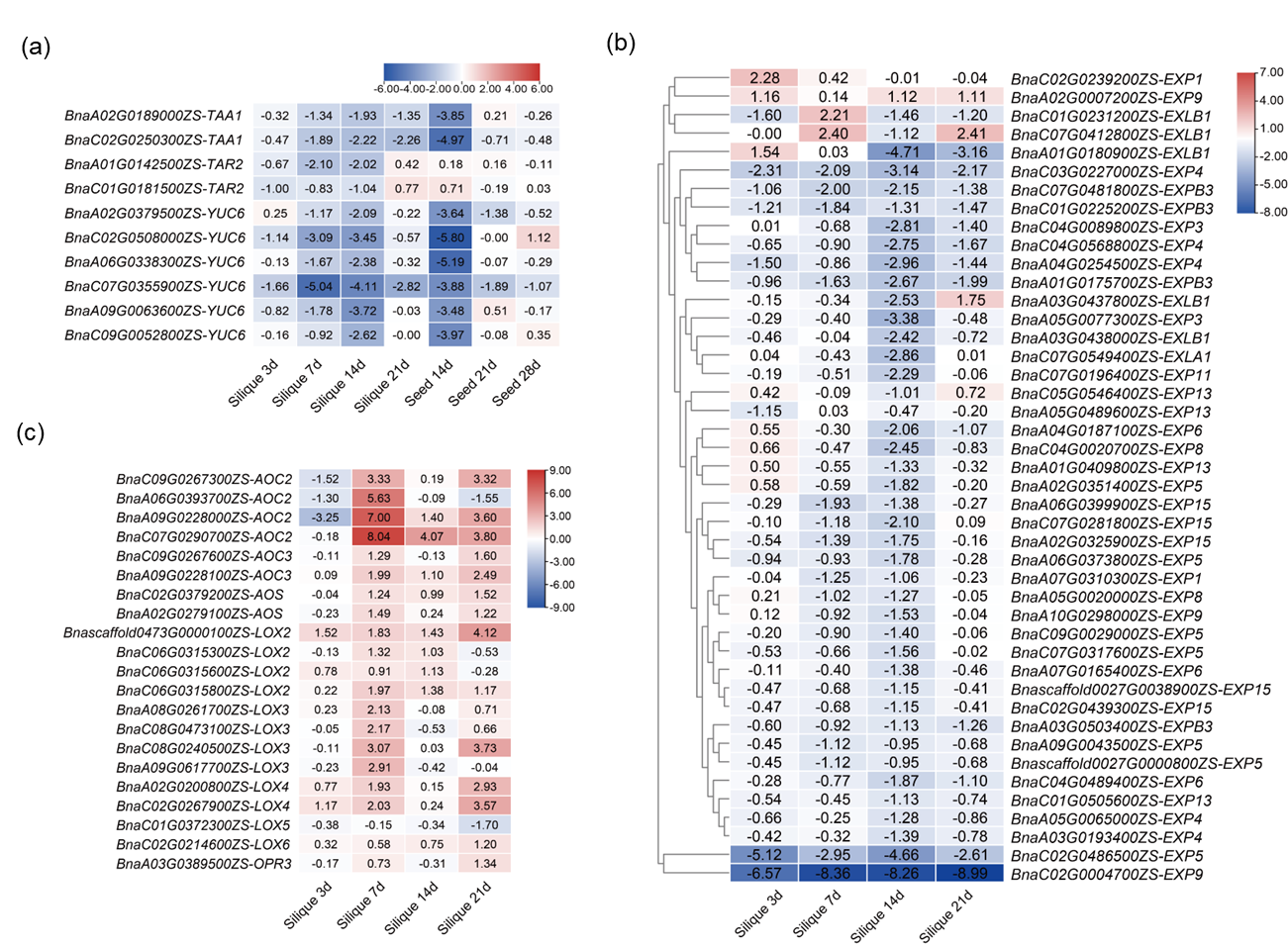


**Supplement figure 6 The expression heatmaps for auxin biosynthesis genes, *BnaEXP* genes, and JA biosynthesis genes.**

(a) The expression heatmap illustrates the DEGs associated with auxin biosynthesis, including *BnaTAA1/TAR2s* and *BnaYUC6s,* in both silique walls and seeds. (b) DEGs of the *BnaEXP* family in silique walls. (c) DEGs of JA biosynthesis genes, including *BnaLOXs*, *BnaAOSs*, *BnaAOCs* and *BnaOPR3*, in silique walls. The numbers within the boxes represent the log2 fold changes when comparing NIL-*ssl* to NIL-9D290, with colors indicate the fold change.


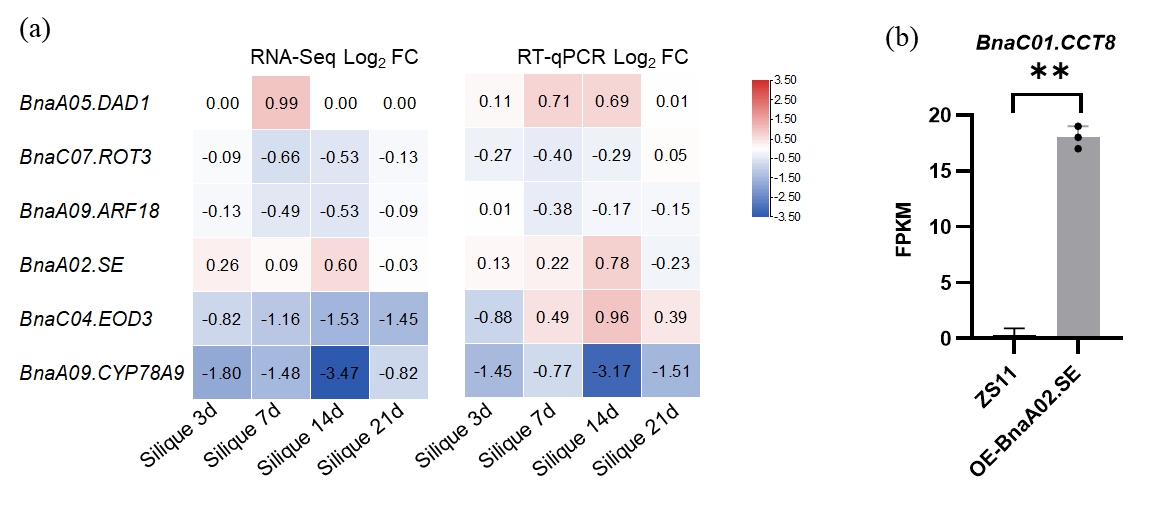


**Supplement figure 7 The expression of several known SL-related genes in silique development.**

(a) The expression heatmap illustrates several known SL-related genes in silique walls, including *BnaA05.DAD1*, *BnaC7.ROT3*, *BnaA09.ARF18*, *BnaA02.SE*, and *BnaEOD3s* and *BnaA09.CYP78A9*. The numbers within the boxes represent the log2 fold changes when comparing NIL-*ssl* to NIL-9D290 derived from RNA-seq data (left) and qRT-PCR analysis (right). *BnaC02.ACTIN7* was used as a control. **, *p* < 0.01. (b) FPKM of *BnaC01.CCT8* in siliques of ‘ZS11’ and *BnaA02.SE2* overexpression lines (Zhang et al. 2024). **, *p* < 0.01.


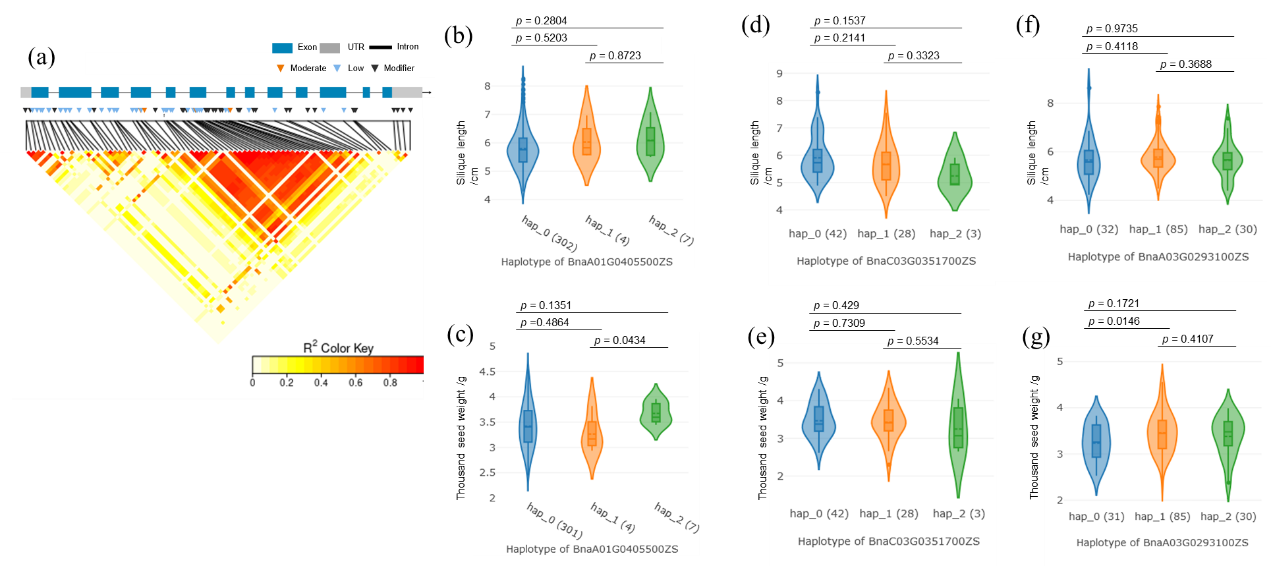


**Supplement figure 8 Analysis of variation information and haplotype of homologous genes of *BnaC01.CCT8*.**

(a) The 81 SNPs are distributed across introns, exons, and both the 5’ and 3’ UTRs. Linkage disequilibrium (LD) analysis of SNPs within *BnaC01.CCT8* genes. Data are from *BnVIR*. (b-g) SL and TSW of homologs of *BnaC01.CCT8*, including *BnaA01G0405500ZS* (b, c)*, BnaA03G0293100ZS* (d, e)*,* *BnaC03G0351700ZS* (f, g). The number of accessions in different haplotypes used for phenotyping are shown. Comparisons relied on ANOVA. Data are from *BnVIR.*


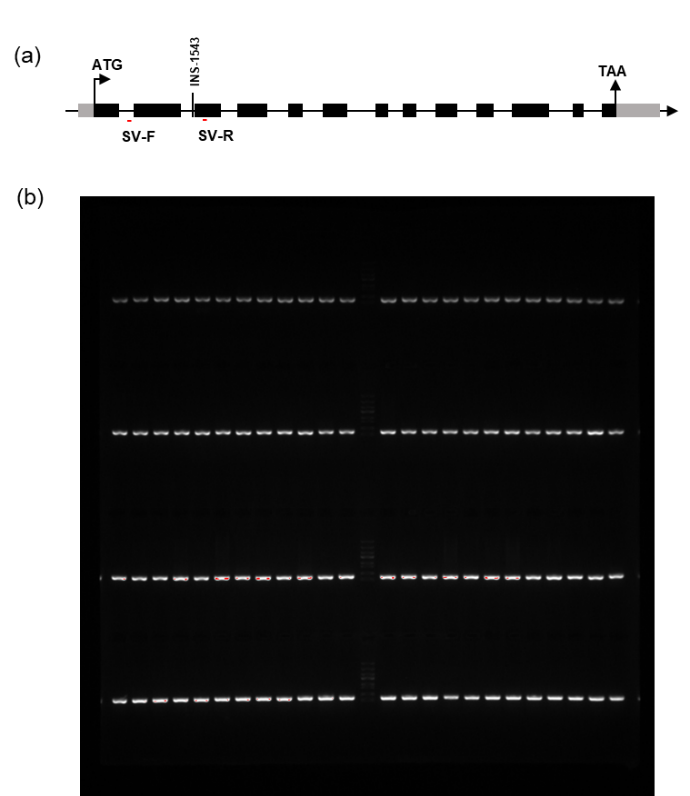


**Supplement figure 9 PCR identification of structural variants (SV).**

(a) The positions of the SV-F and SV-R primers used for the identification of INS-1543. The red line denotes the locations of the SV-F and SV-R primers. (b) Agarose gel analysis of INS-1543 on 96 randomly selected samples from our inbred accessions of *Brassica napus*.


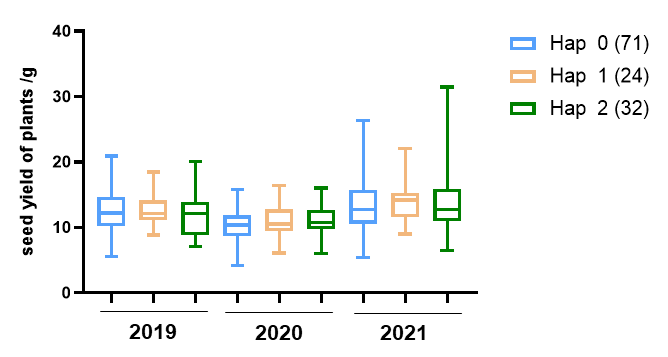


**Supplement figure 10** **Haplotype analysis of *BnaC01.CCT8* in seed yield of plants.**

Seed yield of plants of *BnaC01.CCT8* haplotypes in 127 inbred accessions of *B. napus*. 2019, 2020, and 2021 represents the seed yield of plants and silique per plant data collected from three years, respectively. Number in parentheses indicates the number of lines corresponding to this haplotype.


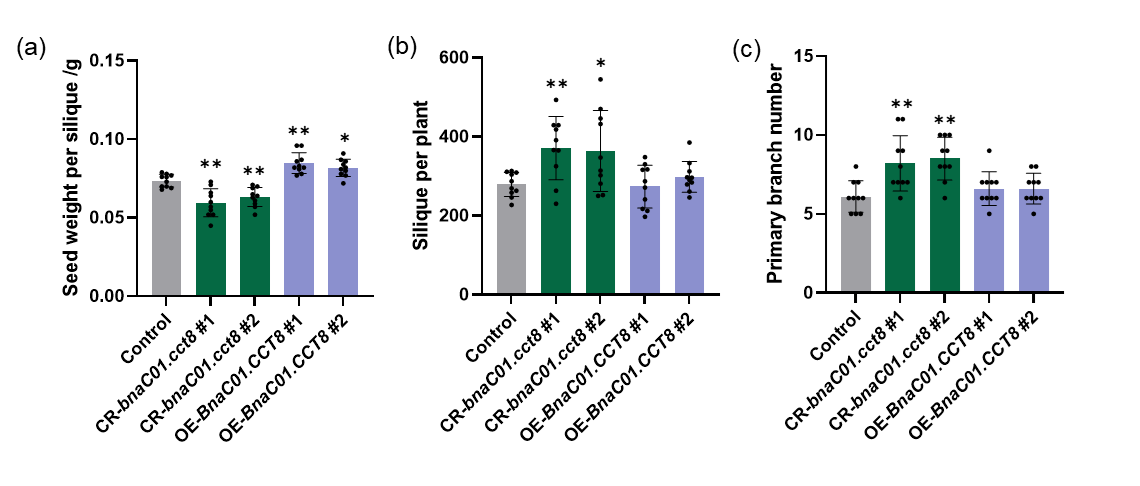


**Supplement figure 11** **Changes of various yield-related traits of gene-edited and overexpressed lines of *BnaC01.CCT8* at maturity in the field.**

(a-c) Seed weight per silique (a), silique per plant (b), and primary branch number (c) for the control (Westar), CRISPR/Cas9-edited lines (CR-*bnac01.cct8*) and overexpression lines (OE-*BnaC01.CCT8*) at maturity. *, *p* < 0.05; **, *p* < 0.01.
